# Supplementary material for: Academic stress and its psychosocial and behavioral determinants in medical students: Findings from a cross-sectional study
Source: PLoS One. 2026 Apr 16;21(4):e0347306. doi: 10.1371/journal.pone.0347306 (PMC13086342; doi:10.1371/journal.pone.0347306)
Supplement: S1 Appendix — (PDF) [file pone.0347306.s001.pdf]

S1: Structural Equation Modeling of Psychological Distress, Behavioral Mediators, and Academic Stress with Sociodemographic Adjustments

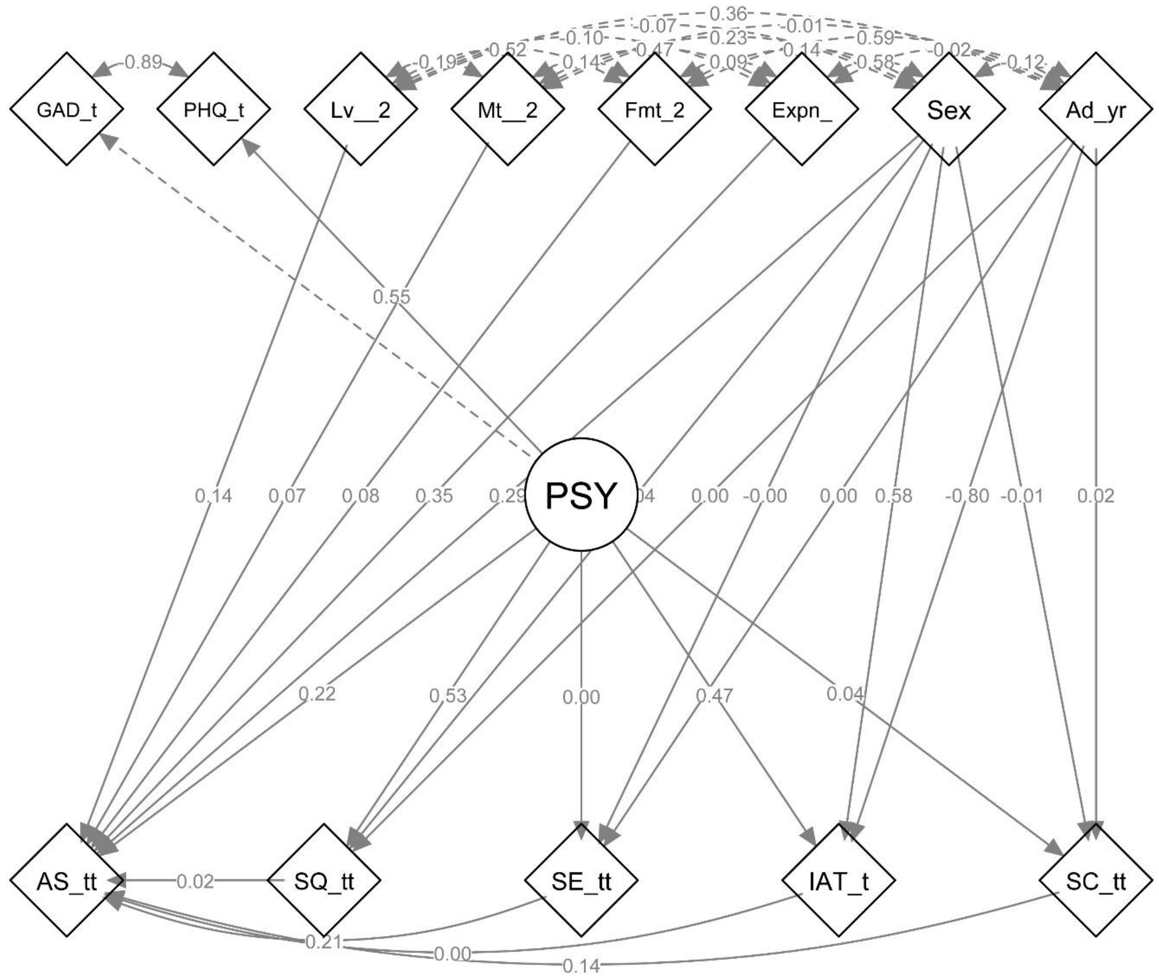

S1 Fig 1: Extended SEM model including sociodemographic factors

Structural equation model (SEM) in which the latent construct PSY (psychological distress; indicators GAD_total = anxiety and PHQ_total = depression) predicts AS_total (academic stress) directly and indirectly via behavioral mediators (SQ_total = sleep disturbance, SE_total = self-esteem, IAT_total = internet addiction, SC_total = stress coping). Sample size: $N = 1{,}072$.

Estimator: WLSMV; missing data handled pairwise. Standard error: robust for indirect effects. Standardized path coefficients: PSY → AS_total = 0.22; PSY → SQ_total = 0.53; PSY → IAT_total = 0.35; PSY → SE_total = 0.00; PSY → SC_total = 0.04. Direct effects of mediators on AS_total: SE_total = 0.22; SC_total = 0.15; SQ_total = 0.02; IAT_total ≈ 0.00. Indirect effects: via

SQ = 0.01; via SC = 0.01; total indirect = 0.02; total effect (direct + indirect) = 0.34 ($\beta \approx 0.24$). Sociodemographic covariates (living area, mother's education, family type, expenditure, sex, and academic year) are included as exogenous controls. Curved dashed lines represent correlations among exogenous variables.

Note: Global fit indices are not reported for models with covariates. Coefficients reflect conditional associations and do not imply causation.
