# Supplementary material for: Academic stress and its psychosocial and behavioral determinants in medical students: Findings from a cross-sectional study
Source: PLoS One. 2026 Apr 16;21(4):e0347306. doi: 10.1371/journal.pone.0347306 (PMC13086342; doi:10.1371/journal.pone.0347306)
Supplement: S2 Appendix — (PDF) [file pone.0347306.s002.pdf]

S2: Comparison of Stress Coping Styles: Segregated vs. Parsimonious Structural Equation Modeling (SEM)

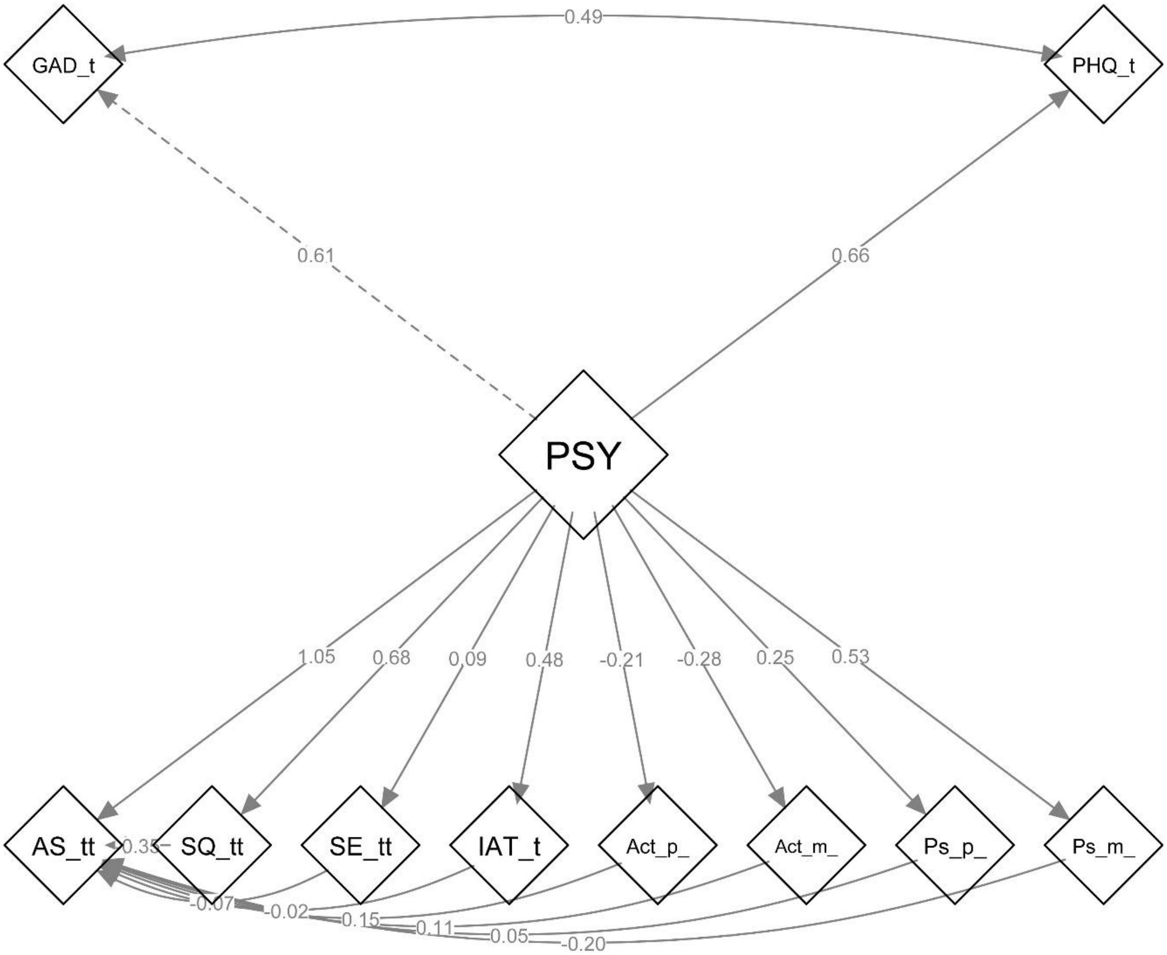

S2 Fig 1: Latent-only Stress Coping Style Inventory (SCSI) segregated SEM model

Tables (1.1, 1.2, 1.3, 1.4): Model fit; Absolute and relative fit measures and fit indices for the Latent-only Stress Coping Style Inventory (SCSI) segregated SEM model

S2 Table 1.1

Model tests			
Label	X^2	df	p
User Model	105.84	9	<.001
Baseline Model	2209.15	21	<.001
Scaled User	167.31	9	<.001
Scaled Baseline	1563.55	21	<.001

S2 Table 1.2

Fit indices					
			95% Confidence Intervals		
Type	SRMR	RMSEA	Lower	Upper	RMSEA p
Classical	0.07	0.10	0.09	0.11	<.001
Robust	0.07	0.11	0.10	0.11	<.001
Scaled	0.07	0.12	0.11	0.13	<.001

S2 Table 1.3

User model versus baseline model

	Model	Scaled	Robust
Comparative Fit Index (CFI)	0.90	0.80	0.90
Tucker-Lewis Index (TLI)	0.83	0.67	0.83
Bentler-Bonett Non-normed Fit Index (NNFI)	0.83	0.67	0.83
Relative Noncentrality Index (RNI)	0.90	0.80	0.90
Bentler-Bonett Normed Fit Index (NFI)	0.89	0.79	
Bollen's Relative Fit Index (RFI)	0.82	0.65	
Bollen's Incremental Fit Index (IFI)	0.90	0.80	
Parsimony Normed Fit Index (PNFI)	0.53	0.47	

S2 Table 1.4

Additional fit indices

	Model
Hoelter Critical N (CN), α=0.05	127.13
Hoelter Critical N (CN), α=0.01	148.67
Goodness of Fit Index (GFI)	1.00
Adjusted Goodness of Fit Index (AGFI)	1.00
Parsimony Goodness of Fit Index (PGFI)	0.41
McDonald Fit Index (MFI)	0.86
Expected Cross-Validation Index (ECVI)	0.39
Loglikelihood user model (H0)	.
Loglikelihood unrestricted model (H1)	.
Akaike (AIC)	.
Bayesian (BIC)	.
Sample-size adjusted Bayesian (SABIC)	.

S2 Table 1.5: R-squared

R^2

Variable	R^2
GAD_total	0.38
PHQ_total	0.43
**AS_total**	**0.55**
SQ_total	0.46
SE_total	0.01
IAT_total	0.23
Act_prob_cop	0.04
Act_emo_cop	0.08
Pas_prob_cop	0.06
Pas_emo_cop	0.28

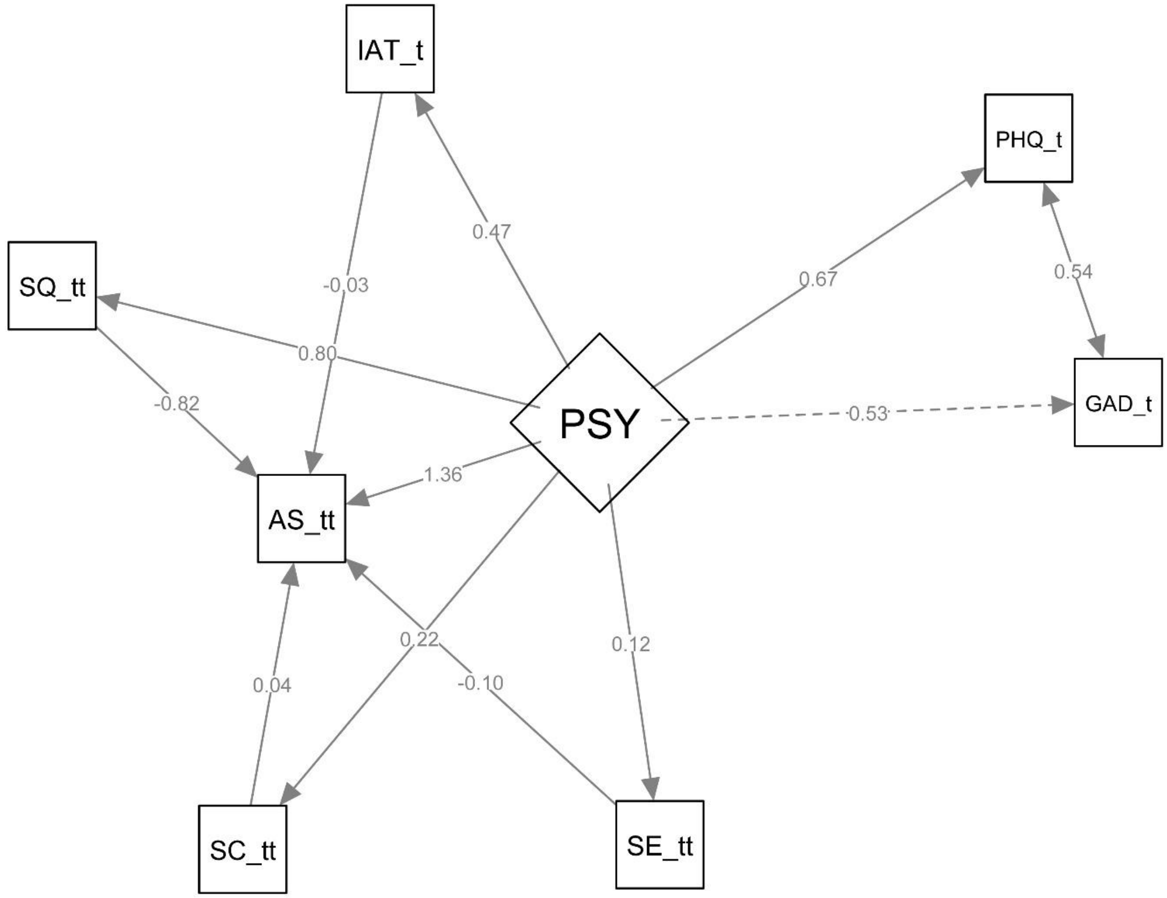

S2 Fig 2: Latent-only Stress Coping Style Inventory (SCSI) parsimonious SEM model

Tables (1.6, 1.7, 1.8, 1.9, 1.10): Model fit; Absolute and relative fit measures and fit indices for the Stress Coping Style Inventory (SCSI) parsimonious SEM model

S2 Table 1.6

Model tests			
Label	X^2	df	p
User Model	105.84	9	<.001
Baseline Model	2209.15	21	<.001
Scaled User	167.31	9	<.001
Scaled Baseline	1563.55	21	<.001

S2 Table 1.7

Fit indices					
			95% Confidence Intervals		
Type	SRMR	RMSEA	Lower	Upper	RMSEA p
Classical	0.05	0.10	0.08	0.12	<.001
Robust	0.05	0.10	0.09	0.12	<.001
Scaled	0.05	0.13	0.11	0.15	<.001

S2 Table 1.8

User model versus baseline model			
	Model	Scaled	Robust
Comparative Fit Index (CFI)	0.96	0.90	0.96
Tucker-Lewis Index (TLI)	0.90	0.76	0.90
Bentler-Bonett Non-normed Fit Index (NNFI)	0.90	0.76	0.90
Relative Noncentrality Index (RNI)	0.96	0.90	0.96
Bentler-Bonett Normed Fit Index (NFI)	0.95	0.89	
Bollen's Relative Fit Index (RFI)	0.89	0.75	
Bollen's Incremental Fit Index (IFI)	0.96	0.90	
Parsimony Normed Fit Index (PNFI)	0.41	0.38	

S2 Table 1.9

Additional fit indices	
	Model
Hoelter Critical N (CN), α=0.05	172.20
Hoelter Critical N (CN), α=0.01	220.24
Goodness of Fit Index (GFI)	1.00
Adjusted Goodness of Fit Index (AGFI)	1.00
Parsimony Goodness of Fit Index (PGFI)	0.26
McDonald Fit Index (MFI)	0.96
Expected Cross-Validation Index (ECVI)	0.15
Loglikelihood user model (H0)	.
Loglikelihood unrestricted model (H1)	.
Akaike (AIC)	.
Bayesian (BIC)	.
Sample-size adjusted Bayesian (SABIC)	.

S2 Table 1.10: R-squared

R^2	
Variable	R^2
GAD_total	0.28
PHQ_total	0.45
AS_total	0.72
SQ_total	0.64
SE_total	0.02
IAT_total	0.22
SC_total	0.05

A comparative evaluation of the segregated SEM model and the parsimonious SEM model revealed notable differences in model fit, explanatory power, and overall efficiency.

Chi-square (χ^2) and Model Test

Both models demonstrated identical chi-square statistics ($\chi^2 = 105.84$, df = 9, $p < .001$), indicating that neither model achieved a perfect fit. However, given the known sensitivity of χ^2 to sample size, greater emphasis was placed on alternative fit indices.

Absolute Fit Indices (SRMR, RMSEA)

The parsimonious model showed improved residual fit with a lower SRMR (0.05 vs. 0.07).
RMSEA values were comparable but remained slightly elevated in both models (~0.10–0.13), suggesting moderate fit.
Confidence intervals for RMSEA were marginally narrower in the parsimonious model.
Interpretation: The parsimonious model demonstrates better residual-based fit, although both models indicate room for improvement.

Incremental / Comparative Fit Indices (CFI, TLI, NFI, IFI, RNI)

The parsimonious model consistently outperformed the segregated model:

- CFI: 0.96 vs. 0.90
- TLI: 0.90 vs. 0.83
- NFI: 0.95 vs. 0.89
- IFI: 0.96 vs. 0.90
- RNI: 0.96 vs. 0.90

Interpretation: The parsimonious model achieves excellent comparative fit (≥0.95), while the segregated model reflects only acceptable fit (~0.90).

Parsimony and Efficiency Indices (PNFI, PGFI, ECVI)

Segregated model: Higher PNFI (0.53 vs. 0.41) and PGFI (0.41 vs. 0.26)
Parsimonious model: Much lower ECVI (0.15 vs. 0.39), indicating better generalizability and predictive performance
Higher Hoelter's CN (172–220 vs. 127–148), indicating greater sample adequacy
Interpretation: The segregated model is more parsimonious structurally,

But the parsimonious model is superior in predictive stability and cross-validation.

Goodness-of-Fit Indices (GFI, AGFI, MFI)

Both models showed perfect GFI and AGFI (1.00), likely due to model characteristics.
The parsimonious model had substantially higher MFI (0.96 vs. 0.86), indicating better overall fit quality.

Explained Variance (R^2 Comparison)

The parsimonious model demonstrated substantially greater explanatory power, particularly for key outcomes:

Academic Stress (AS_total): 0.72 vs. 0.55
Sleep Quality (SQ_total): 0.64 vs. 0.46
Depression (PHQ_total): Slightly higher (0.45 vs. 0.43)
Interpretation: The parsimonious model provides stronger explanatory power for primary outcomes, especially academic stress.

Conclusion:

Although both models demonstrated comparable chi-square statistics, the parsimonious SEM model exhibited superior performance across multiple fit indices, including lower SRMR, higher CFI, TLI, and IFI, as well as improved cross-validation metrics (ECVI). Importantly, it explained a greater proportion of variance in key outcomes such as academic stress and sleep quality. While the segregated model showed slightly better parsimony indices (PNFI, PGFI), the parsimonious model offered a more robust, generalizable, and theoretically coherent representation of the data. Therefore, the parsimonious model was retained as the final model for interpretation.
