# Supplementary material for: Academic stress and its psychosocial and behavioral determinants in medical students: Findings from a cross-sectional study
Source: PLoS One. 2026 Apr 16;21(4):e0347306. doi: 10.1371/journal.pone.0347306 (PMC13086342; doi:10.1371/journal.pone.0347306)
Supplement: S3 Appendix — [Sociodemographic variables not included]. (PDF) [file pone.0347306.s003.pdf]

S3 file: Latent-only variables network and centrality graph. [Sociodemographic variables not included]

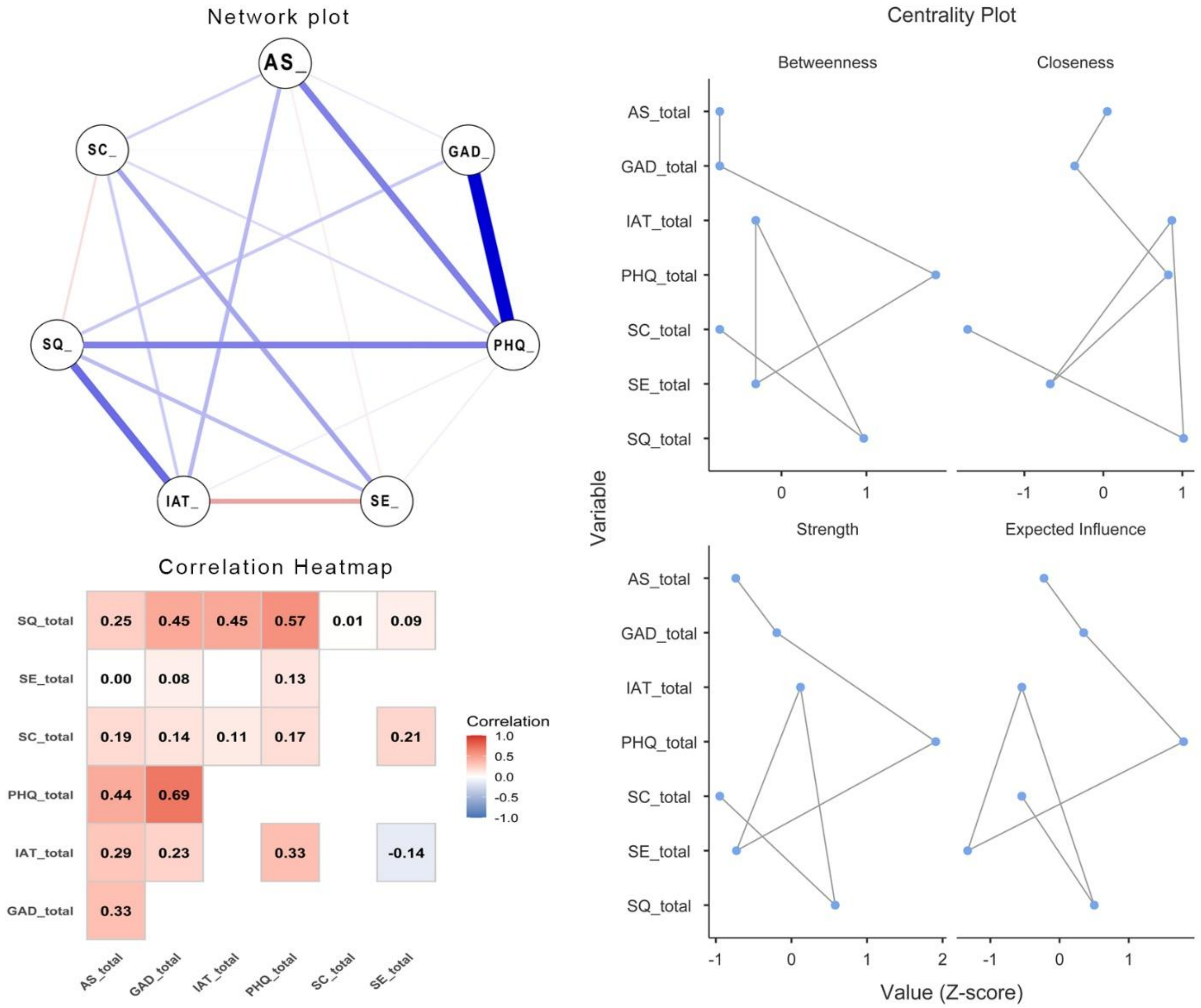

S3 Fig 1: Network Structure and Centrality of Psychological and Behavioral Measures

Caption: Network analysis of seven measures (AS total, GAD_total, PHQ_total, SC_total, SE_total, SQ_total, IAT_total) showing conditional associations (top left), pairwise Pearson correlations (heatmap, bottom left), and node centrality metrics (right panels: Betweenness, Closeness, Strength, Expected Influence). Edges in the network are colored and weighted by sign and magnitude; thicker blue edges indicate stronger positive conditional associations. Depressive symptoms (PHQ_total) and generalized anxiety (GAD_total) are the most strongly connected and

most central nodes; sleep quality (SQ_total) shows a moderate positive correlation with PHQ_total (≈0.57); internet addiction (IAT_total) and self-efficacy (SE_total) show a weak negative correlation (≈−0.14). Network edges represent conditional relationships estimated from the sample and do not imply causation.

The figure *(Supplementary Figure 3)* **shows** a network of psychological and behavioral variables, a correlation heatmap of their pairwise associations, and four centrality measures (Betweenness, Closeness, Strength, and Expected Influence) that quantify each variable's importance in the network.

Variables: AS_total (Academic stress), GAD_total (Anxiety), PHQ_total (Depression), SC_total (Stress coping), SE_total (Self-esteem), SQ_total (Sleep quality, IAT_total (Internet addiction).

Key relationships (network)

- **PHQ_total — GAD_total:** strongest positive link; thick, dark blue edge indicates a large positive association.
- **SQ_total — PHQ_total:** moderately strong positive association (correlation ≈ **0.57**).
- **IAT_total — SE_total:** weak negative association (correlation ≈ **−0.14**); thin red edge.
- **Other edges:** several moderate positive connections among anxiety, sleep, and social constructs, with edge thickness and color reflecting magnitude and sign.

Correlation heatmap highlights

- **Largest positive correlations:** PHQ_total with GAD_total (**0.69**); PHQ_total with SQ_total (**0.57**).
- **Smallest/negative correlations:** IAT_total with SE_total (**−0.14**).
- **Range and pattern:** correlations span from modest negative to strong positive, suggesting clustered symptom domains (mood/anxiety/sleep) and weaker links with internet-addiction and self-efficacy.

Centrality summary

- **Highest centrality (most influential nodes):** variables with the greatest Strength and Expected Influence are likely **PHQ_total** and **GAD_total**, indicating they sit at the core of the network and connect strongly to multiple nodes.
- **Bridging roles:** variables with elevated Betweenness may act as connectors between clusters (likely **SQ_total** or **SC_total** depending on plot heights).

- **Peripheral nodes:** variables with low Strength and Expected Influence (likely **IAT_total** and **SE_total**) are less central and have weaker direct influence on the network.

Interpretation and implications

- **Clinical/behavioral meaning:** depressive symptoms (PHQ) and generalized anxiety (GAD) are tightly coupled and drive much of the network structure; sleep problems (SQ) are strongly associated with depressive symptoms, suggesting sleep may be an important intervention target.
- **Targets for intervention:** focusing on highly central nodes (PHQ, GAD, and possibly SQ) could produce broader downstream effects across the network.
- **Caveats:** network edges reflect conditional associations in the sample and do not prove causation; centrality depends on estimation method and sample characteristics; small correlations (e.g., −0.14) are weak and should be interpreted cautiously.
