# Supplementary material for: Academic stress and its psychosocial and behavioral determinants in medical students: Findings from a cross-sectional study
Source: PLoS One. 2026 Apr 16;21(4):e0347306. doi: 10.1371/journal.pone.0347306 (PMC13086342; doi:10.1371/journal.pone.0347306)
Supplement: S4 Appendix — (PDF) [file pone.0347306.s004.pdf]

S4 file: Network and centrality analysis of psychological, behavioral, and sociodemographic variables related to academic stress.

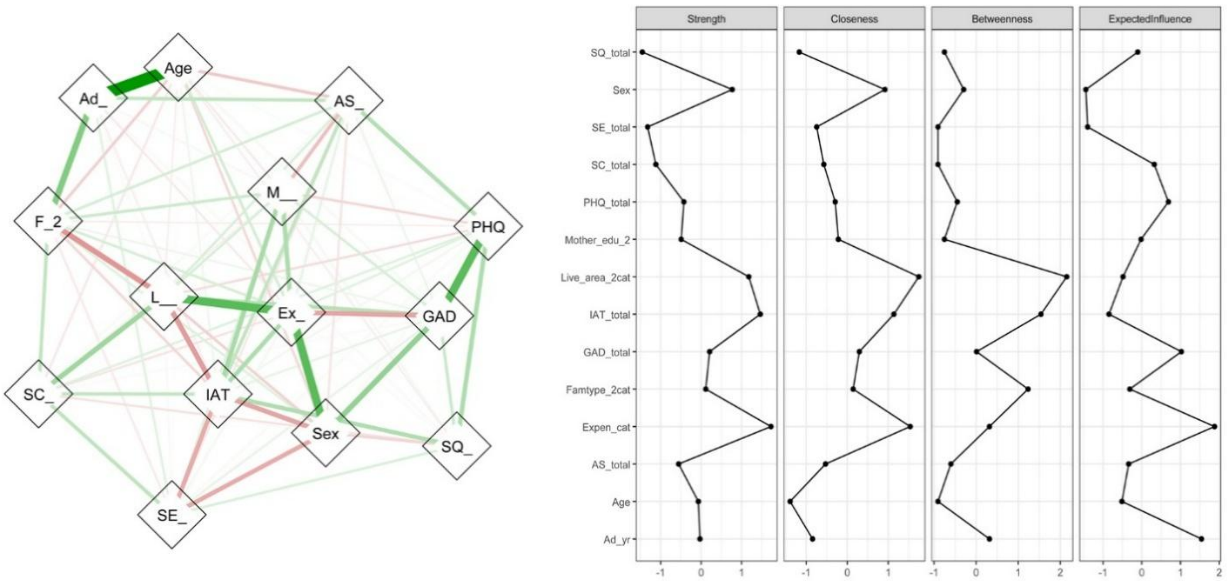

S4 Fig 1

S4 Table 1: Partial correlation

Correlation matrix - Spearman

	PHQ_total	GAD_total	AS_total	SE_total	IAT_total	SC_total	SQ_total	Age
PHQ_total	—							
GAD_total	0.72***	—						
AS_total	0.44***	0.36***	—					
SE_total	0.10**	0.11***	-0.06*	—				
IAT_total	0.35***	0.28***	0.34***	-0.20***	—			
SC_total	0.17***	0.14***	0.16***	0.20***	0.11***	—		
SQ_total	0.57***	0.51***	0.28***	0.06*	0.45***	0.03	—	
Age	-0.06*	-0.03	-0.22***	0.10***	0.02	-0.04	-0.05	—

Note. : * p < .05, ** p < .01, *** p < .001

Figure Caption: Left panel: regularized partial-correlation network of study variables (nodes = observed variables; edges = conditional associations controlling for all other nodes). Edge color indicates direction (green = positive; red = negative), and thickness indicates magnitude. Right

panels: standardized centrality indices (Strength, Closeness, Betweenness, Expected Influence). Variables included: psychological measures (PHQ_total, GAD_total), behavioral measures (SQ_total, SE_total, SC_total, IAT_total), academic stress (AS_total), and sociodemographic covariates [Age, Ad_yr (Admission year), Expen_cat (Average monthly expenditure categories), Live_area_2cat (Living area categories), Mother_edu_2 (Mothers' education categories), Famtype_2cat (Family type categories, Sex). Sample: $N = 1{,}072$.

Partial Correlations (Supplementary Table 3): Spearman rank correlations were used, and a robust correlation estimate was computed using the percentage method. Network estimation: graphical lasso (glasso) with EBIC model selection (EBICglasso; $\gamma = 0.5$, default) was applied to the robust Spearman correlation matrix to obtain a sparse partial-correlation network; visualization produced with qgraph. Robustness and inference: edge-weight confidence intervals and centrality stability (CS) coefficients were estimated using non-parametric bootstrap procedures with 5,000 resamples; report edge-weight bootstrap CIs and CS-coefficients in the manuscript. Key empirical values (Spearman): PHQ–GAD = 0.72 ($p < .001$); PHQ–SQ = 0.57 ($p < .001$); PHQ–AS = 0.44 ($p < .001$); IAT–SE = −0.20 ($p < .001$); IAT–PHQ = 0.35 ($p < .001$). Centrality summary: IAT_total, Expen_cat, and Live_area_2cat show comparatively high Strength and Expected Influence, indicating these nodes act as important connectors between psychological, behavioral, and sociodemographic domains. Caveat: edges are regularized partial correlations conditional on all other nodes and depend on estimation choices; they do not establish causality.
