# Supplementary material for: Academic stress and its psychosocial and behavioral determinants in medical students: Findings from a cross-sectional study
Source: PLoS One. 2026 Apr 16;21(4):e0347306. doi: 10.1371/journal.pone.0347306 (PMC13086342; doi:10.1371/journal.pone.0347306)
Supplement: Attachment — Submitted filename: 1_Plos One_maunscript.docx [file pone.0347306.s005.docx]

Academic Stress and Its Psychosocial and Behavioral Determinants in Medical Students: Findings from a Cross-Sectional Study

Md Rizwanul Karim^1^*, S. A. Sazin Haque^2^, Faiza Rumeen^3^, Purna Aruneema^4^

Affiliation

^1^Associate Professor (Epidemiology), Department of Community Medicine, Shaheed M. Monsur Ali Medical College (SMMAMC), Sirajganj, Postal code: 6700, Bangladesh.

Email: [shameem.m25@gmail.com](mailto:shameem.m25@gmail.com)

[https://orcid.org/](https://orcid.org/%20) 0000-0003-2922-9290

^2^Research Assistant, Department of Medicine, Patuakhali Medical College, Patuakhali. Postal Code: 8600, Bangladesh.

Email: [sazinhaque4@gmail.com](mailto:sazinhaque4@gmail.com)

^3^Research Assistant, Department of Medicine, Patuakhali Medical College, Patuakhali. Postal Code: 8600, Bangladesh.

Email: [faizarumeen@gmail.com](mailto:faizarumeen@gmail.com)

^4^Research Assistant, Department of Medicine, Patuakhali Medical College, Patuakhali. Postal Code: 8600, Bangladesh

Email: [purna.aruneema@gmail.com](mailto:purna.aruneema@gmail.com)

***Corresponding author: Md Rizwanul Karim^1^,** [**Shameem.m25@gmail.com**](mailto:Shameem.m25@gmail.com)

**Abstract**

Background: Academic stress is a widespread challenge among medical students, shaped by a complex interplay of sociodemographic, psychological, and behavioral factors. This study aims to explore how personal, psychological, and behavioral factors influence academic stress.

Methods: Using a probability sampling technique, a cross-sectional survey was conducted between October and December 2022 among 1,072 medical students from eight public colleges in Bangladesh. The Academic Stress Scale (ASS-40), was utilized to assess academic stress, while the Patient Health Questionnaire (PHQ-9), Generalized Anxiety Disorder (GAD-7), Insomnia Severity Index (ISI), Internet Addiction Test (IAT), Stress Coping Style Inventory (SCSI), and Rosenberg Self-Esteem Scale (RSES) were employed to evaluate depression, anxiety, insomnia, internet addiction, coping strategies, and self-esteem, respectively. Logistic regression identified significant predictors, reported as adjusted odds ratios (AOR) with 95% confidence intervals (CI). Kindly mentioned about what are the statistical test used for analysis the data.

Results: This study highlights the complex and multifaceted nature of academic stress among medical students, identifying significant sociodemographic, psychological, and behavioral predictors. Findings suggest that urban residency, higher paternal education, minority status, and specific living arrangements are associated with elevated levels of academic stress. Moreover, psychological factors, particularly severe depression, anxiety, and insomnia, contribute significantly to stress levels. Maladaptive behaviors such as internet addiction and ineffective coping styles were found to exacerbate stress; however, passive emotional coping provided a modest protective effect.

Conclusion: Academic stress among medical students is multifaceted and driven by sociocultural, psychological, and behavioral factors. To alleviate stress, targeted interventions such as mental health support programs, parental awareness initiatives, and training on coping strategies should be implemented.

**Keywords**: **Academic-Stress, Depression, Coping, Anxiety, Insomnia, Internet Addiction.**

**Introduction**

Academic stress is a widespread issue among medical students globally, shaped by a complex interplay of psychological, behavioral, and sociodemographic factors. The demanding nature of medical education, characterized by long study hours, high academic expectations, and rigorous assessments, often leads to significant stress among students. Academic stress, if unattended, can adversely affect students' academic performance, mental health, and physical well-being, resulting in burnout, depression, and anxiety (1, 2). Medical students are particularly vulnerable to stress-related challenges due to the intense and competitive nature of their educational environment (3). In recent years, there has been growing recognition of the mental health challenges faced by medical students, which has encouraged extensive research into the prevalence and determinants of academic stress (4, 5).

Psychological factors such as anxiety, depression, and insomnia are well-documented contributors to academic stress among medical students (4-6). Depression has been identified as a major predictor of academic stress. Studies have consistently highlighted a high prevalence of depressive symptoms among medical students, which exacerbates stress levels (7, 8). Behavioral factors, including eating behavior, internet addiction, and coping strategies, also play a significant role in shaping stress levels (9, 10). A study conducted in Egypt found that internet addiction increases academic stress among undergraduate students by negatively affecting their time management and study habits (11). Moreover, ineffective coping mechanisms, such as passive emotional coping, have been associated with increased stress levels (12).

Sociodemographic factors add complexity to the issue of academic stress. Research shows that variables such as parental education, religion, and living arrangements significantly affect stress levels among medical students (4, 13). For instance, students from urban areas generally report higher stress levels than their rural counterparts (13, 14). Additionally, parental education, especially maternal education, plays a crucial role; students whose mothers have lower educational qualifications tend to experience more stress, likely due to socioeconomic challenges and limited emotional support (13). Furthermore, students living with family often report higher stress levels than those living with friends, indicating that family dynamics may also contribute to their stress (15).

Despite extensive research on academic stress among medical students, notable gaps persist. Without integrating these variables into a broader framework, most studies examine isolated factors such as anxiety, parental background, and psychological traits. In particular, the influence of behavioral patterns, including maladaptive coping strategies and excessive internet use, remains underexplored (16, 17). The relative impact of sociodemographic, psychological, and behavioral factors remains unclear, especially when considered collectively. Much of the existing research has been conducted in Western contexts, leaving a limited understanding of how unique sociocultural factors shape academic stress in other regions, particularly in South Asia. To address the identified gaps in the literature, this study adopts a cross-sectional psychometric survey design targeting medical students within a representative academic setting. The primary aim is to examine how various sociodemographic, psychological, and behavioral factors are associated with academic stress. In particular, the study will assess the influence of variables such as parental education, religion, and living arrangements, along with psychological conditions including depression, anxiety, and insomnia. Furthermore, it will investigate the impact of behavioral aspects, particularly internet addiction and coping mechanisms, on students' stress levels.

**Method and Sampling:**

This cross-sectional study was conducted with medical students from eight public medical colleges across the eight divisions of Bangladesh, between October 1 and December 30, 2022. A simple random sampling technique was utilized to secure adequate representation from five academic years and all eight medical colleges. The required sample size was calculated using the <https://epitools.ausvet.com.au/samplesize> sample size calculator based on the following parameters: a total population size (N) of 57,100 medical students (Yearly enrollment of approximately 11,420 students per year across five academic years), a hypothesized outcome proportion (p) of 54% (18), a margin of error (d) of ±3% (0.03), a 95% confidence level (Z = 1.96), and a design effect (DEFF) of 1, assuming a simple random sampling design. The formula used for sample size estimation was: n = [DEFF × N × p × (1−p)] / [(d² / Z²) × (N−1) + p × (1−p)].

The sample size calculation yielded an estimated requirement of approximately 1,042 participants. To accommodate a potential 5% nonresponse rate, the target sample size was adjusted to around 1,094. Each medical college consisted of five academic years, ranging from first year to fifth year, and from these, 28 students were randomly selected each academic session. This process resulted in 140 students per college, totaling 1,120 participants across the eight institutions. To ensure sufficient statistical power and to compensate for potential data loss, a total of 1,120 students were recruited. Data were collected using a self-administered structured questionnaire. After data cleaning to remove incomplete or inconsistent responses, 1,072 fully completed questionnaires were included in the final analysis.

**Data Collection and Research Instrument:**

Before starting the study, we obtained ethical approval from the Institutional Review Board and secured written informed consent from all participants. We arranged for administrative support through a formal request submitted to the college principal, which included a briefing session to explain the study protocol to participants. For data collection, we used a pretested, structured questionnaire in Bengali. This questionnaire was translated and back-translated by bilingual experts to ensure linguistic and conceptual accuracy. The study employed a comprehensive questionnaire comprising 132 items, organized into eight sections that captured socio-demographic details and incorporated several validated assessment tools. The average completion time was approximately 25 minutes. The questionnaire featured a range of established self-report instruments measuring academic stress, coping mechanisms, mental health status, self-esteem, internet usage, and sleep quality. All tools used in the survey exhibited strong psychometric validity and had been previously adapted for Bengali-speaking populations where applicable. Socio-demographic variables included age, gender, type of residence (urban or rural), relationship status, parental education, household income, and religious affiliation.

**Academic Stress Scale (ASS)**: A 40-item instrument originally developed by Kim (1970) and later adapted for the Indian context by Rajendran & Kaliappan (1990), measures five dimensions of academic stress: personal inadequacy, fear of failure, interpersonal difficulties with teachers, teacher-pupil relationship/teaching methods, and inadequate study facilities. Items are rated on a 5-point Likert scale (0 = "No Stress" to 4 = "Extreme Stress"). The overall score can reach a maximum of 160, and a threshold score of 67.13 is established to determine significant academic stress. The scale shows strong reliability (Cronbach’s α = 0.70; test-retest reliability = 0.82) (19).

**Stress Coping Style Inventory (SCSI)**: Adapted from Lin & Chen (2010), this 28-item scale evaluates four coping styles: active emotional, passive emotional, active problem, and passive problem coping. Items are rated from 1 ("Completely disagree") to 5 ("Completely agree"), yielding total scores between 28 and 140. The instrument demonstrated excellent reliability, with Cronbach’s alpha ranging from 0.860 to 0.876 across subscales, and 0.830 for the overall scale (20).

**Patient Health Questionnaire-9 (PHQ-9)**: This 9-item tool assesses depressive symptoms over the previous two weeks, with responses rated on a 0–3 scale. Total scores range from 0 to 27, with standard cut-offs for depression severity (e.g., 5–9 for mild, 10–14 for moderate). The Bengali version used in this study has shown strong internal consistency (Cronbach’s α = 0.837) and good split-half reliability (0.848) (21).

**Generalized Anxiety Disorder-7 (GAD-7)**: A 7-item screening tool for anxiety symptoms, rated from 0 ("Not at all") to 3 ("Nearly every day"). Total scores range from 0 to 21, with a cut-off of 10 indicating clinically significant anxiety. The Bengali adaptation has excellent reliability (Cronbach’s α = 0.895) and demonstrated good convergent validity in university populations (22).

**Rosenberg Self-Esteem Scale (RSES)**: This 10-item scale measures global self-esteem using a 4-point Likert scale (0 = "Strongly Disagree" to 3 = "Strongly Agree"), yielding scores from 0 to 30. Scores below 15 indicate low self-esteem. The scale has high internal consistency (Cronbach’s α = 0.86) (23).

**Internet Addiction Test (IAT)**: A 20-item instrument developed to assess internet addiction severity, covering four domains: lack of control, social withdrawal/emotional conflict, time management issues, and concealment of problematic behavior. Items are rated on a 6-point Likert scale (0 = "Does not apply" to 5 = "Always"), with scores ranging from 0 to 100. The scale demonstrates strong psychometric properties, with a Cronbach’s alpha between 0.85 and 0.91, and explains 56.5% of the total variance (24).

**Insomnia Severity Index (ISI)**: A 7-item scale assessing insomnia symptoms and their impact, with responses on a 0–4 scale. Total scores range from 0 to 28; scores ≥15 indicate clinically significant insomnia. The ISI has shown excellent reliability (Cronbach’s α = 0.90–0.91) and strong convergent validity. A cut-off score of 10 optimally distinguishes insomnia cases with high sensitivity (86.1%) and specificity (87.7%) (25).

**Statistical analysis:** The data were cleaned and analyzed using the Windows versions of SPSS 23.0 and Jamovi 2.6.25. Participants were divided into two groups based on a cutoff score of 67 on the Academic Stress Scale—those experiencing academic stress and those not. Sociodemographic characteristics included sex, age, parental education, residence, personal income, and marital status, while psychological factors encompassed anxiety, depression, insomnia, and self-esteem. Behavioral factors included internet addiction and academic stress coping. Descriptive and inferential data were presented through tables and graphs, utilizing parametric (t-test) and non-parametric (χ²) methods to assess relationships among the variables. Effect sizes were determined using Phi, Cramer's V, and Cohen's d. A binary logistic regression model was created to identify significant predictors, controlling for confounders, with model validity assessed through standard metrics. Regression results were reported as beta coefficients and Adjusted Odds Ratios (AOR) with 95% confidence intervals and p-values.

**Results**

This cross-sectional study revealed significant associations between academic stress and various sociodemographic, psychological, and behavioral factors. Most participants were female (61.8%, n = 663), though academic stress levels did not significantly differ between males and females. The majority of students were Muslim (87.4%, n = 937), while Hindu students (12.6%, n = 135) reported notably higher levels of academic stress. In terms of parental education, 47.0% (n = 504) of mothers had completed SSC or equivalent, while 93.0% (n = 997) of fathers had attained at least HSC-level education. Students whose mothers had less than SSC and those whose fathers had HSC or higher qualifications experienced higher stress levels. Most students were from urban areas (85.8%, n = 921), and academic stress was more prevalent among urban than rural students. Regarding living arrangements, the majority lived with peers (73.3%, n = 786), yet those residing with family (21.4%, n = 229) reported higher stress. Similarly, dormitory residents (60.2%, n = 645) experienced lower stress compared to those living outside dormitories. Nearly all participants were unmarried (96.6%, n = 1,036), and marital status was not associated with academic stress. Financial dependence was common, with 84.5% (n = 906) reporting no personal income, indicating potential economic vulnerability among students. [Table 1].

**Table 1. Distribution of Academic Stress Levels by Sociodemographic Factors**

| Characteristics | Categories | Academic stress | | Total |  | | ***Phi/***  ***Cramér's V*** |
| --- | --- | --- | --- | --- | --- | --- | --- |
|  |  | Absent n (%) | Preset n (%) | N (%) | ***χ2(df)*** | ***p-value*** |  |
| **Sex** | *Male* | 202 (49.4) | 207 (50.6) | 409 (100) | 2.399 (1) | 0.121 | 0.049^*^ |
|  | *Female* | 361 (54.4) | 302 (45.6) | 663 (100) |  |  |  |
| **Religion** | *Islam* | 531 (56.7) | 406 (43.3) | 937 (100) | 51.427 (1)^ψ^ | 7.43e^-13^ | 0.219^*^ |
|  | *Hinduism* | 32 (23.7) | 103 (76.3) | 135 (100) |  |  |  |
| **Mother’s Education** | *Below SSC* | 31 (31) | 69 (69) | 100 (100) |  |  |  |
|  | *SSC or Equivalent* | 281 (55.8) | 223 (44.2) | 504 (100) | 20.918 (2)^ψ^ | 2.87e^-5^ | 0.140^**^ |
|  | *HSC and above or equivalent* | 251 (53.6) | 217 (46.4) | 468 (100) |  |  |  |
| **Father’s Education** | *Below SSC* | 54 (84.4) | 10 (15.6) | 64 (100) |  |  |  |
|  | *SSC or Equivalent* | 0 (0) | 11 (100) | 11 (100) | 39.071 (2) ^€^ | 1.58e^-10^ | 0.191^**^ |
|  | *HSC and above or equivalent* | 509 (51.1) | 488 (48.9) | 997 (100) |  |  |  |
| **Personal income** | *No* | 485 (53.5) | 421 (46.5) | 906 (100) | 2.154 (1) | 0.142 | 0.047^*^ |
|  | *Yes* | 78 (47.0) | 88 (53.0) | 166 (100) |  |  |  |
| **Living area** | *Rural* | 93 (61.2) | 59 (38.8) | 152 (100) | 4.936 (1)^ψ^ | 0.026 | 0.071^*^ |
|  | *Urban* | 470 (51.1) | 450 (48.9) | 920 (100) |  |  |  |
| **Living with** | *Friends* | 449 (57.1) | 337 (42.9) | 786 (100) | 25.065 (1)^ψ^ | 7.92e^-7^ | 0.153^*^ |
|  | *Family* | 114 (39.9) | 172 (60.1) | 286 (100) |  |  |  |
| **Living in Dormitory** | *No* | 8 (15.7) | 43 (84.3) | 51 (100) | 27.601(1)^ψ^ | 1.49e^-7^ | 0.165^*^ |
|  | *Yes* | 555 (54.4) | 466 (45.6) | 1021 (100) |  |  |  |
| **Marital status** | *Unmarried* | 544 (52.5) | 492 (47.5) | 1036 (100) | .000 (1) | 1.000 | 0.001^*^ |
|  | *Married* | 19 (52.8) | 17 (47.2) | 36 (100) |  |  |  |

^ψ^ *The Chi-square test indicates a statistically significant result at the 0.05 level,*

**Phi statistic for chi-square test &** Cramer's v for chi-square test,*

€*The Fisher’s exact test indicates a statistically significant result at the 0.05 level*

According to the findings, 52.52% of participants indicated they did not experience academic stress, whereas 47.48% reported that they did. Anxiety levels varied, with many reporting mild (35.07%) or severe (25.19%) anxiety. Higher academic stress was linked to increased anxiety, as 61.5% of students with severe anxiety faced academic stress compared to 30.9% with minimal anxiety. A strong association was also found between academic stress and depression, with 90.2% of severely depressed students experiencing stress compared to 26.2% with minimal depression. Insomnia showed a similar pattern, with 63.8% of those with moderate insomnia reporting stress versus 41.0% without significant insomnia. Internet addiction was another factor, as 70.0% of students with moderate addiction reported stress compared to 33.3% with no addiction. All students with severe internet addiction experienced academic stress. Coping strategies varied; stressed students relied more on passive-emotional and problem-focused coping strategies, while active emotional coping was lower. Active problem-focused coping showed no significant difference between groups. The analysis of mean scores for psychiatric and behavioral factors across different categories of academic stress showed that students experiencing academic stress exhibited significantly higher levels of anxiety, depression, insomnia, and internet addiction compared to those without stress [Figure 1, Table 2]. Additionally, a clear positive correlation was found between the severity of depression and academic stress scores. Students with severe or moderately severe depression reported the highest levels of stress, particularly concerning inadequate study facilities and fear of failure. In contrast, students with minimal or no depression reported the lowest stress levels across all areas [Figure 2].

Figure 1. **Box-violin plots showing the distribution of depression, anxiety, insomnia, and internet addiction scores among participants with and without academic stress. Each plot illustrates the median, interquartile range, and score distribution density. Comparisons highlight differences in psychological outcomes based on academic stress experience**.

Table 2. **Psychological and Behavioral Factors of Academic Stress among Medical Students**

| **Characteristics** | **Categories** | | | | **Academic stress** | | **Total** |  |  | ***Phi/***  ***Cramér's V*** | |
| --- | --- | --- | --- | --- | --- | --- | --- | --- | --- | --- | --- |
|  |  |  |  |  | **Absent n (%)** | **Preset n (%)** | **N (%)** | ***χ2 (df)*** | ***p-value*** |  |  |
| **Anxiety** | *Minimal* | | | | 125 (69.1) | 56 (30.9) | 181 (100) |  |  |  | |
|  | *Mild* | | | | 236 (62.8) | 140 (37.2) | 376 (100) | 72.315 (3)^ψ^ | 1.36e^-15^ | 0.260*^**^* | |
|  | *Moderate* | | | | 98 (40) | 147 (60) | 245 (100) |  |  |  | |
|  | *Severe* | | | | 104 (38.5) | 166 (61.5) | 270 (100) |  |  |  | |
| **Depression** | *None/minimal* | | | | 169 (73.8) | 60 (26.2) | 229(100) |  |  |  | |
|  | *Mild* | | | | 239 (67.5) | 115 (32.5) | 354 (100) | 179.617 (4)^ψ^ | 8.16e^-38^ | 0.410*^**^* | |
|  | *Moderate* | | | | 104 (38.8) | 164 (61.2) | 268 (100) |  |  |  | |
|  | *Moderately severe* | | | | 43 (30.9) | 96 (69.1) | 139 (100) |  |  |  | |
|  | *Severe* | | | | 8(9.8) | 74 (90.2) | 82 (100) |  |  |  | |
| **Insomnia** | *No clinically significant* | | | | 239(59.0) | 166 (41.0) | 405 (100) |  |  |  | |
|  | *Subthreshold* | | | | 226 (55.8) | 179 (44.2) | 405 (100) | 33.035 (3)^ψ^ | 3.17e^-7^ | 0.176*^**^* | |
|  | *Clinically significant moderately severe* | | | | 71 (36.2) | 125(63.8) | 196 (100) |  |  |  | |
|  | *Clinically significant severe* | | | | 27 (40.1) | 39 (50.9) | 66 (100) |  |  |  | |
| **Internet addiction** | *Not significant* | | | | 336(66.7) | 168 (33.3) | 504 (100) |  |  |  | |
|  | *Mild* | | | | 154 (48.0) | 167 (52.0) | 321 (100) | 96.772 (3) ^€^ | 9.19e^-22^ | 0.300*^**^* | |
|  | *Moderate* | | | | 73 (30.0) | 170 (70.0) | 243 (100) |  |  |  | |
|  | *Severe* | | | | 0 (0) | 4 (100) | 4 (100) |  |  |  | |
| **Self-esteem** | *Low* | | | | 157 (51.3) | 149 (48.7) | 306 (100) | .252 (1) | .616 | 0.015*^*^* | |
|  | *Normal to high* | | | | 406 (53) | 360 (47) | 766 (100) |  |  |  | |
|  | | **N** | | | **Mean (SD)** | |  | **Standard error of**  **Difference** |  |  | |
| **Total scores of psychological and behavioral factors** | | **Academic stress absent** | **Academic stress present** | | **Academic stress Absent** | **Academic stress Present** | **Mean difference** |  | **t** | ***p-value*** | ***Cohen's d*** |
| Anxiety | | 563 (52.52) | | 509 (47.48) | 8.631(5.100) | 11.566 (5.567) | -2.935 | 0.326 | -9.010*ᵃ* | 9.27e^-19^ | -0.551 |
| Depression | | 563 (52.52) | | 509 (47.48) | 7.572 (4.703) | 12.155 (6.208) | -4.583 | 0.335 | -13.701*ᵃ* | 1.74e^-39^ | -0.838 |
| Insomnia | | 563 (52.52) | | 509 (47.48) | 9.185 (5.840) | 11.371 (6.314) | -2.187 | 0.371 | -5.890*ᵃ* | 5.16e^-9^ | -0.360 |
| IAT | | 563 (52.52) | | 509 (47.48) | 28.725 (17.506) | 40.674 (18.682) | -11.949 | 1.105 | -10.809 | 6.41e^-26^ | -0.661 |
| Self-esteem | | 563 (52.52) | | 509 (47.48) | 16.055 (2.737) | 15.945 (2.469) | 0.110 | 0.160 | 0.689 | 0.491 | 0.042 |
| Active emotional coping | | 563 (52.52) | | 509 (47.48) | 29.448(4.367) | 28.880 (4.221) | 0.567 | 0.263 | 2.158 | 0.031 | 0.132 |
| Passive emotional coping | | 563 (52.52) | | 509 (47.48) | 15.094 (4.332) | 16.212 (4.031) | -1.118 | 0.256 | -4.361*ᵃ* | 1.42e^-5^ | -0.267 |
| Active problem coping | | 563 (52.52) | | 509 (47.48) | 21.355 (3.034) | 21.279 (3.360) | 0.076 | 0.195 | 0.390 | 0.696 | 0.024 |
| Passive problem coping | | 563 (52.52) | | 509 (47.48) | 21.030 (3.640) | 22.028 (3.965) | -0.997 | 0.232 | -4.293*ᵃ* | 1.92e^-5^ | -0.263 |

Ψ *The Chi-square test indicates a statistically significant result at the 0.05 level*

**Phi statistic for chi-square test,*

***Cramer's v for chi-square test*

*λ Cohen’s d for t-test*

€*The Fisher’s exact test indicates a statistically significant result at the 0.05 level*

*ᵃ Levene's test is significant (p < .05), suggesting a violation of the assumption of equal variances*

**Figure 2. Mean Academic Stress Sub-scales Scores Across Depression Severity Categories**

The logistic regression model analyzed predictors of academic stress among medical students, using academic stress (present vs. absent) as the dependent variable. Key independent variables included sociodemographic, psychological, behavioral, and socioeconomic factors. The model showed a good-fit with a deviance of 1033.53 and an Akaike Information Criterion (AIC) of 1091.53. Pseudo-R² values indicated moderate to strong explanatory power (McFadden's = 0.30, Cox and Snell's = 0.34, Nagelkerke's = 0.46, Tjur's = 0.36), with a classification accuracy of 76%, specificity of 82%, sensitivity of 70%, and an area under the curve (AUC) of 0.84.

Sociodemographic findings revealed that students living with friends had a 2.6-fold increased likelihood of experiencing academic stress (AOR = 2.623, 95% CI: 1.760–3.910). Urban students were 3.04 times more likely to face academic stress than rural students (AOR = 3.038, 95% CI: 1.803–5.121). Higher maternal education reduced the likelihood of stress (AOR = 0.032, 95% CI: 0.013–0.079), while higher paternal education significantly increased it (AOR = 25.806, 95% CI: 8.344–79.811). Hindu students had a 3.57 times greater likelihood of experiencing stress than Muslim students (AOR = 3.568, 95% CI: 1.959–6.499). Psychological factors indicated that students with severe depression had a 35.71 times higher probability of experiencing academic stress (AOR = 35.707, 95% CI: 12.439–102.504). Moderate anxiety was associated with a 2.25 times higher likelihood of stress (AOR = 2.254, 95% CI: 1.214–4.185). Notably, sub-threshold and clinically moderate insomnia correlated with reduced likelihood of academic stress (AOR = 0.420 and 0.247, respectively).

**Table 3.** **Sociodemographic, Psychological, and Behavioral Predictors of Academic Stress**

| **Predictors** | Collinearity statistics | | B | S.E. | Wald | df | Sig. | Crude odds ratio | 95% C.I.for EXP(B) | | Adjusted odds ratio | 95% C.I.for EXP(B) | |
| --- | --- | --- | --- | --- | --- | --- | --- | --- | --- | --- | --- | --- | --- |
|  | VIF | Tolerance |  |  |  |  |  |  | Lower | Upper |  | Lower | Upper |
| Living with; Family – Friends* | 1.95 | 0.913 | 0.964 | .204 | 22.446 | 1 | .000 | 2.010 | 1.526 | 2.648 | 2.623 | 1.760 | 3.910 |
| Living area; Urban – Village/rural* | 1.155 | 0.866 | 1.111 | .266 | 17.407 | 1 | .000 | 1.509 | 1.063 | 2.144 | 3.038 | 1.803 | 5.121 |
| Mother’s Education; SSC – Below SSC* | 1.320 | 0.757 | -2.587 | .428 | 36.475 | 1 | .000 | 0.357 | 0.225 | 0.564 | 0.075 | 0.032 | 0.174 |
| Mother’s Education; =>HSC – Below SSC* | 1.320 | 0.757 | -3.435 | .456 | 56.734 | 1 | .000 | 0.388 | 0.245 | 0.616 | 0.032 | 0.013 | 0.079 |
| Father’s Education; =>HSC – Below SSC* | 1.204 | 0.831 | 3.251 | .576 | 31.843 | 1 | .000 | 5.177 | 2.607 | 10.281 | 25.806 | 8.344 | 79.811 |
| Religion; Hinduism – Islam* | 1.051 | 0.951 | 1.272 | .306 | 17.293 | 1 | .000 | 4.989 | 3.204 | 7.770 | 3.568 | 1.959 | 6.499 |
| Depression; Moderate – Minimal* | 1.192 | 0.839 | 1.129 | .292 | 14.930 | 1 | .000 | 4.442 | 3.027 | 6.518 | 3.094 | 1.745 | 5.487 |
| Depression; Moderately severe – Minimal* | 1.192 | 0.839 | 1.455 | .343 | 17.947 | 1 | .000 | 6.288 | 3.950 | 10.010 | 4.285 | 2.186 | 8.401 |
| Depression; Severe – Minimal* | 1.192 | 0.839 | 3.575 | .538 | 44.157 | 1 | .000 | 26.054 | 11.865 | 57.213 | 35.707 | 12.439 | 102.504 |
| Anxiety; Moderate – Minimal* | 1.236 | 0.809 | 0.813 | .316 | 6.624 | 1 | .010 | 3.348 | 2.231 | 5.124 | 2.254 | 1.214 | 4.185 |
| Insomnia; Subthreshold Insomnia – No* | 1.235 | 0.809 | -0.868 | .226 | 14.809 | 1 | .000 | 0.481 | 0.283 | 0.816 | 0.420 | 0.270 | 0.653 |
| Insomnia; Clinical moderate Insomnia – No* | 1.235 | 0.809 | -1.400 | .318 | 19.342 | 1 | .000 | 0.548 | 0.323 | 0.930 | 0.247 | 0.132 | 0.460 |
| Internet use; Mild Internet use – No* | 1.190 | 0.840 | 0.678 | .213 | 10.142 | 1 | .001 | 2.169 | 1.628 | 2.889 | 1.970 | 1.298 | 2.990 |
| Internet use; Moderate Internet use – No* | 1.190 | 0.840 | 2.386 | .290 | 67.788 | 1 | .000 | 4.658 | 3.345 | 6.485 | 10.872 | 6.161 | 19.187 |
| Passive emotional coping | 1.338 | 0.747 | -0.060 | .024 | 6.367 | 1 | .012 | 1.066 | 1.035 | 1.097 | 0.941 | 0.898 | 0.987 |

Note. Estimates represent the log odds of "Academic stress = Present" vs. "Academic stress = Absent"

Model fit measures: Deviance = 1033.53, AIC = 1091.53, McFadden's pseudo-R-squared = .30, Cox and Snell’s R^2^ = .34, Nagelkerke’s R^2^= .46, Tjur’s R^2^ = .36.

Variance inflation factor VIF range = [ 1.05 to 1.34] less than 4

Tolerance range = [.75 to .95] greater than .25

Case classification summary; Accuracy = .76, Specificity = .82, Sensitivity = .70, Area Under Curve AUC = .84

* Reference category

SSC = Secondary School Certificate, HSC = Higher Secondary School Certificate

In terms of behavioral factors, moderate internet use was linked to a 10.87-fold increase in stress risk (AOR = 10.872, 95% CI: 6.161–19.187), while passive emotional coping slightly reduced the likelihood of stress (AOR = 0.941, 95% CI: 0.898–0.987). [Table 3].

Researchers created a forest plot to illustrate the primary predictors of academic stress using the results from a logistic regression model. The log10-transformed plot reinforces the regression findings, clearly identifying the most significant predictors, as each confidence interval (CI) remains above or below the null line at 0, corresponding with p-values under 0.05. Notably, the predictors with the highest effect sizes are Depression (comparing severe to minimal) and Father’s Education (comparing ≥HSC to below SSC), which are positioned furthest to the right, indicating their substantial adjusted odds ratios (AORs) of 35.707 (Log10(35.707) = 1.553) and 25.806 (Log10(25.806) = 1.4117), respectively. In contrast, Mother’s Education (comparing ≥HSC to below SSC) and the comparison of SSC to below SSC appear farthest to the left, reflecting their strong protective effects with AORs of 0.032 (Log10(0.032) = -1.495) and 0.075 (Log10(0.075) = -1.125). [Figure 3].

**Figure 3: Binary Logistic Regression-Based Forest Plot of Significant Predictors of Academic Stress Using Log₁₀-Transformed AORs**

**Discussion**

This study underscores the complex interplay of sociodemographic, psychological, and behavioral factors contributing to academic stress among medical students. Although consistent with global research, our findings offer distinct perspectives apposite to the South Asian context, highlighting the diverse origins and critical drivers of academic stress.

Sociodemographic factors were found to have a significant relationship with academic stress experienced by medical students. Urban students were three times more likely to experience stress than their rural peers, aligning with studies that associate urban living with increased academic competition and social pressure (26). Living arrangements also played a key role: students residing with family reported higher stress levels than those living with friends, likely due to greater household responsibilities and family expectations. Moreover, students not living in dormitories experienced significantly more stress, possibly due to the absence of structured peer support and academic resources typically available in campus housing. This contrasts with previous studies that indicated students living away from home experienced more stress, particularly those experiencing language barriers (4, 27). Our research, however, points to urban living and family situations as more significant factors contributing to stress. Religious background also turned out to be a notable predictor, as students from minority groups reported experiencing higher levels of stress. This supports existing literature linking minority status and perceived discrimination with mental health challenges, including academic stress and burnout (28). Parental education showed a complex relationship with academic stress. Consistent with findings from a study in Pakistan (16), students from disadvantaged backgrounds, particularly those living at home, outside dormitories, or from minority religious groups, faced elevated stress levels. While Eames et al. (29) emphasized challenges faced by underrepresented or first-generation students in the U.S., our South Asian context highlighted maternal education as a protective factor, potentially due to increased emotional support and academic guidance. Interestingly, students whose fathers had higher education levels were 25 times more likely to experience stress, possibly reflecting elevated parental expectations and academic pressure. This mirrors findings from a recent Indian study that identified parental expectations and living arrangements as major contributors to stress among medical students (30).

The study identified significant associations between psychological factors and academic stress. Severe depression emerged as the strongest predictor, with affected students being 35 times more likely to experience academic stress, consistent with prior research highlighting the bidirectional link between mental health disorders and academic distress in adolescents and college students (4, 7). Moderate to severe anxiety also increased the likelihood of academic stress, supporting existing evidence of anxiety’s role in contributing to academic difficulties (31). Behavioral factors such as internet addiction and insomnia were also found to be important, though often overlooked. Insomnia was significantly associated with academic stress, echoing findings that poor sleep impairs cognitive function, emotional regulation, and stress resilience (32). Interestingly, subthreshold and moderate insomnia showed a slight protective effect, possibly due to increased wakefulness and study time, an observation that warrants further investigation. Internet addiction was another strong predictor, with students exhibiting moderate addiction levels being about 10 times more likely to report academic stress. This aligns with studies showing that excessive internet use negatively affects time management, study habits, and academic performance (33, 34).

Coping strategies played a complex role. Passive emotional coping was associated with slightly lower levels of academic stress, contradicting some research that links maladaptive coping with heightened stress responses (35). Nonetheless, both passive emotional and passive problem-focused coping were significantly more common among stressed students. These avoidant strategies, such as denial, disengagement, procrastination, and avoidance, are known to worsen stress by failing to address its root causes and are linked to poor psychological outcomes (12, 36, 37). In contrast, active emotional coping, including techniques like seeking emotional support and cognitive reappraisal, was modestly associated with lower stress. These strategies support emotional regulation and resilience and have been shown to reduce psychological distress among students (36, 38). The effect size was small, indicating that while active emotional coping can be helpful, it might not alone be sufficient to alleviate academic stress.

The study found no significant differences in self-esteem or active problem-focused coping scores between students facing academic stress and those who are not. While active coping strategies like time management and goal setting are often associated with improved academic performance, their effectiveness in reducing stress varies based on individual traits and academic demands (37). The results suggest that active coping strategies might not be universally effective in high-pressure academic situations. Logistic regression analysis revealed that passive emotional coping provided a modest protective effect against academic stress after adjusting for confounding variables. Although this may seem counterintuitive, the short-term relief offered by such strategies likely helps students temporarily disengage from stressors and restore emotional balance (39). However, prolonged dependence on passive emotional coping can hinder effective stress management and potentially worsen academic challenges over time (40). Promoting adaptive emotional coping while reducing maladaptive responses may help mitigate academic stress among students.

**Strengths and Limitations**

This research has notable strengths, including a large random sample from evenly distributed institutions, which enhances the generalizability of the findings. It utilized validated psychometric tools and advanced multivariate logistic regression for meticulous assessment of academic stress and its associated factors. The inclusion of recent literature further adds to its relevance. However, limitations exist. The cross-sectional design restricts causal inferences, and self-reported data may be affected by recall or social desirability bias. Although key confounders were considered, factors like personality traits, institutional or social support, and resilience were not addressed.

**Conclusion and Recommendations**

This study highlights the complex interplay of factors contributing to academic stress among medical students, with higher stress levels linked to urban background, paternal education, minority status, and specific living conditions. Psychological issues like depression, anxiety, and insomnia, along with maladaptive behaviors such as internet addiction and ineffective coping styles, were found to exacerbate stress. To address this, medical institutions should adopt a comprehensive approach that includes mental health support, promotion of healthy lifestyles, parental involvement, training in effective coping strategies, and sustained screening efforts.

**List of abbreviations:**

AOR (Adjusted Odds Ratio)

CI (Confidence Interval)

Academic Stress Scale (ASS)

Stress Coping Style Inventory (SCSI)

Patient Health Questionnaire-9 (PHQ-9)

Generalized Anxiety Disorder-7 (GAD-7)

Rosenberg Self-Esteem Scale (RSES)

Internet Addiction Test (IAT)

Insomnia Severity Index (ISI)

SPSS (Statistical Package for the Social Sciences)

**Declarations**

### **Ethical statement:**

This survey received ethical approval from the Patuakhali Medical College Research Ethics Committee (PkMC-REC-23-05-S19). The study was conducted in full compliance with the Ethical principles of the Declaration of Helsinki, with strict attention to maintaining participants’ privacy, confidentiality, and anonymity. Before data collection, participants were given a clear explanation of the study’s purpose and informed of their voluntary involvement, including their right to withdraw at any time. Written informed consent was obtained separately using a dedicated consent form, securely attached to the semi-structured, interviewer-administered questionnaire. Participants were assured that all personal information would remain confidential and anonymous, and that the results of the study would be used solely for the public good.

**Consent for publication:**

It is not relevant since no personal information was disclosed other than the unique anonymous identification number assigned to the respondent, and there was no violation of confidentiality.

**Availability of data and materials:**

The data that support the findings of this study are openly available in figshare at

Karim, Md Rizwanul (2025). Psychological, Behavioral, and Socioeconomic Correlates of Stress in Bangladeshi Medical Students: A Cross-Sectional Study. figshare. Dataset. <https://doi.org/10.6084/m9.figshare.29014094.v1>

**Competing interests:**

The authors state that the study was performed without any commercial or financial connections that may be seen as possible conflicts of interest.

**Funding:**

This study was undertaken as part of the academic curriculum for 4th-phase medical students during the Residential Field Site Training (RFST) program. It was conducted independently, without financial assistance from public, private, commercial, or non-profit organizations. Moreover, the authors did not receive any remuneration or honorarium for their involvement in the research.

**Acknowledgments:**

Patuakhali Medical College, Directorate General of Medical Education.

**Authors' contributions:**

**Authors' information:**

Md Rizwanul Karim, Associate Professor, Epidemiology; Department of Community Medicine

Shaheed M. Monsur Ali Medical College (SMMAMC), Sirajganj,

Ministry of Health and Family Welfare, Bangladesh.

[Shameem.m25@gmail.com](mailto:Shameem.m25@gmail.com)

<https://orcid.org/0000-0003-2922-9290>

<https://scholar.google.com/citations?user=HeL-gD8AAAAJ>

References

1. Bergmann C, Muth T, Loerbroks A. Medical students' perceptions of stress due to academic studies and its interrelationships with other domains of life: a qualitative study. Med Educ Online. 2019 Dec;24(1):1603526. doi: 10.1080/10872981.2019.1603526. PMID: 31007152; PMCID: PMC6493308.
2. Dyrbye LN, Thomas MR, Shanafelt TD. Systematic review of depression, anxiety, and other indicators of psychological distress among U.S. and Canadian medical students. Acad Med. 2006 Apr;81(4):354-73. doi: 10.1097/00001888-200604000-00009. PMID: 16565188.
3. Suriakumar J, Murugalakshmi T, Sree Bavai Malar B, Devi J. Stress factors among medical students - a survey based analysis of academic and personal challenge. Eur J Cardiovasc Med. 2025 Apr;15(4):436-41. DOI : 10.61336/ejcm/25-04-80
4. Ebrahim OS, Sayed HA, Rabei S, Hegazy N. Perceived stress and anxiety among medical students at Helwan University: A cross-sectional study. J Public Health Res. 2024 Feb 3;13(1):22799036241227891. doi: 10.1177/22799036241227891. PMID: 38313630; PMCID: PMC10838489.
5. Zeng W, Chen R, Wang X, Zhang Q, Deng W. Prevalence of mental health problems among medical students in China: A meta-analysis. Medicine (Baltimore). 2019 May;98(18):e15337. doi: 10.1097/MD.0000000000015337. PMID: 31045774; PMCID: PMC6504335.
6. Vidović S, Rakić N, Kraštek S, Pešikan A, Degmečić D, Zibar L, Labak I, Heffer M, Pogorelić Z. Sleep Quality and Mental Health Among Medical Students: A Cross-Sectional Study. J Clin Med. 2025 Mar 26;14(7):2274. doi: 10.3390/jcm14072274. PMID: 40217724; PMCID: PMC11989636.
7. Fawzy M, Hamed SA. Prevalence of psychological stress, depression and anxiety among medical students in Egypt. Psychiatry Res. 2017 Sep;255:186-194. doi: 10.1016/j.psychres.2017.05.027. Epub 2017 May 17. PMID: 28575777.
8. Dahlin M, Joneborg N, Runeson B. Stress and depression among medical students: a cross-sectional study. Med Educ. 2005 Jun;39(6):594-604. doi: 10.1111/j.1365-2929.2005.02176.x. PMID: 15910436.
9. Melaku L, Bulcha G, Worku D. Stress, anxiety, and depression among medical undergraduate students and their coping strategies. Educ Res Int. 2021 December;9880309. doi:10.1155/2021/9880309.
10. Tang J, Yu Y, Du Y, Ma Y, Zhang D, Wang J. Prevalence of internet addiction and its association with stressful life events and psychological symptoms among adolescent internet users. Addict Behav. 2014 Mar;39(3):744-7. doi: 10.1016/j.addbeh.2013.12.010. Epub 2013 Dec 18. PMID: 24388433.
11. Ali HFM, Mousa MA, Atta MHR, Morsy SR. Exploring the association between internet addiction and time management among undergraduate nursing students. BMC Nurs. 2024 Sep 11;23(1):632. doi: 10.1186/s12912-024-02273-5. PMID: 39256720; PMCID: PMC11389558.
12. Abouammoh N, Irfan F, AlFaris E. Stress coping strategies among medical students and trainees in Saudi Arabia: a qualitative study. BMC Med Educ. 2020 Apr 22;20(1):124. doi: 10.1186/s12909-020-02039-y. PMID: 32321498; PMCID: PMC7178558*.*
13. Yusoff MS, Abdul Rahim AF, Baba AA, Ismail SB, Mat Pa MN, Esa AR. Prevalence and associated factors of stress, anxiety and depression among prospective medical students. Asian J Psychiatr. 2013 Apr;6(2):128-33. doi: 10.1016/j.ajp.2012.09.012. Epub 2012 Nov 23. PMID: 23466109.
14. Elgar FJ, Arlett C, Groves R. Stress, coping, and behavioural problems among rural and urban adolescents. J Adolesc. 2003 Oct;26(5):574-585. doi: 10.1016/s0140-1971(03)00057-5. PMID: 12972270.
15. Vagiri R, Mohlabe K, Mailula L, Nhubunga F, Maepa M, Mphasha M, Mokoena M, Mayimele N, Bangalee V, Grootboom W, Makhele L, Padayachee N. Exploring Anxiety and Depression Among Medical Undergraduates in South Africa: A Cross-Sectional Survey. Healthcare (Basel). 2025 Mar 16;13(6):649. doi: 10.3390/healthcare13060649. PMID: 40150499; PMCID: PMC11941862.
16. Deng Y, Cherian J, Khan NUN, Kumari K, Sial MS, Comite U, Gavurova B, Popp J. Family and Academic Stress and Their Impact on Students' Depression Level and Academic Performance. Front Psychiatry. 2022 Jun 16;13:869337. doi: 10.3389/fpsyt.2022.869337. PMID: 35782431; PMCID: PMC9243415.
17. Al-Dubai SA, Al-Naggar RA, Alshagga MA, Rampal KG. Stress and coping strategies of students in a medical faculty in malaysia. Malays J Med Sci. 2011 Jul;18(3):57-64. PMID: 22135602; PMCID: PMC3216229.
18. Eva EO, Islam MZ, Mosaddek AS, Rahman MF, Rozario RJ, Iftekhar AF, Ahmed TS, Jahan I, Abubakar AR, Dali WP, Razzaque MS, Habib RB, Haque M. Prevalence of stress among medical students: a comparative study between public and private medical schools in Bangladesh. BMC Res Notes. 2015 Jul 30;8:327. doi: 10.1186/s13104-015-1295-5. PMID: 26223786; PMCID: PMC4520268.

*doi:10.4236/ojpsych.2022.122014.*

19. Wahid MH, Sethi MR, Shaheen N, Javed K, Qazi IA, Osama M, Ilah A, Firdos T. Effect of academic stress, educational environment on academic performance & quality of life of medical & dental students; gauging the understanding of health care professionals on factors affecting stress: A mixed method study. PLoS One. 2023 Nov 28;18(11):e0290839. doi: 10.1371/journal.pone.0290839. PMID: 38015910; PMCID: PMC10683982.
20. Lin YM, Chen FS. A stress coping style inventory of students at universities and colleges of technology. World Trans Eng Technol Educ. 2010;8(1):67-72.
21. Naher, R., Rabby, M. R. A., & Sharif, F. (2021). Validation of patient health questionnaire-9 for assessing depression of adults in Bangladesh. Dhaka University Journal of Biological Sciences, 30(2), 275–281. https://doi.org/10.3329/dujbs.v30i2.54652
22. Dhira TA, Rahman MA, Sarker AR, Mehareen J. Validity and reliability of the Generalized Anxiety Disorder-7 (GAD-7) among university students of Bangladesh. PLoS One. 2021 Dec 16;16(12):e0261590. doi: 10.1371/journal.pone.0261590. PMID: 34914811; PMCID: PMC8675645.
23. Akhter MS, Ferdous R. Reliability and validity of the Rosenberg self-esteem scale among university students of Bangladesh. Int J Soc Syst Sci. 2019;11(1):35-50. doi:10.1504/IJSSS.2019.10019219.
24. Samaha AA, Fawaz M, El Yahfoufi N, Gebbawi M, Abdallah H, Baydoun SA, Ghaddar A, Eid AH. Assessing the Psychometric Properties of the Internet Addiction Test (IAT) Among Lebanese College Students. Front Public Health. 2018 Dec 17;6:365. doi: 10.3389/fpubh.2018.00365. PMID: 30619806; PMCID: PMC6305082.
25. Morin CM, Belleville G, Bélanger L, Ivers H. The Insomnia Severity Index: psychometric indicators to detect insomnia cases and evaluate treatment response. Sleep. 2011 May 1;34(5):601-8. doi: 10.1093/sleep/34.5.601. PMID: 21532953; PMCID: PMC3079939.
26. Reddy K. J, Menon K. R, Thattil A. Academic Stress and its Sources Among University Students. Biomed Pharmacol J 2018;11(1). DOI : https://dx.doi.org/10.13005/bpj/1404
27. Gupta S, Choudhury S, Das M, Mondol A, Pradhan R. Factors causing stress among students of a medical college in Kolkata, India. Educ Health (Abingdon). 2015 Jan-Apr;28(1):92-5. doi: 10.4103/1357-6283.161924. PMID: 26261123.
28. Dornisch SJ, Sievert LL, Sharmeen T, Begum K, Muttukrishna S, Chowdhury O, Bentley GR. Religious minority identity associates with stress and psychological health among Muslim and Hindu women in Bangladesh and London. Am J Hum Biol. 2024 Dec;36(12):e24057. doi: 10.1002/ajhb.24057. Epub 2024 Feb 28. PMID: 38415876.
29. Eames D, Thomas S, Norman K, Simanton E, Weisman A. Sociodemographic disadvantage in the burden of stress and academic performance in medical school: implications for diversity in medicine. BMC Med Educ. 2024 Mar 29;24(1):348. doi: 10.1186/s12909-024-05263-y. PMID: 38553726; PMCID: PMC10981295.
30. Chauhan HM, Shah HR, Chauhan SH, Chaudhary SM. Stress in medical students: a cross sectional study. Int J Biomed Adv Res. 2014 Jun;5(6):293-4. doi:10.7439/ijbar. ISSN 2229-3809. Available from:

<https://www.researchgate.net/publication/283464208_Stress_in_medical_students_A_cross_sectional_study>.

31. Alam, M.J., Pratik, M.I.K., Khan, A.H. *et al.* Prevalence and level of stress among final-year students at a health science institute in Bangladesh. *Discov Ment Health* **5**, 9 (2025). https://doi.org/10.1007/s44192-025-00136-2
32. Deb S, Strodl E, Sun J. Academic stress, parental pressure, anxiety and mental health among Indian high school students. Int J Psychol Behav Sci. 2015;5(1):26-34. doi:10.5923/j.ijpbs.20150501.04. Available from: http://journal.sapub.org/ijpbs. License: Creative Commons Attribution 4.0.
33. Kuss DJ, Griffiths MD, Karila L, Billieux J. Internet addiction: a systematic review of epidemiological research for the last decade. Curr Pharm Des. 2014;20(25):4026-52. doi: 10.2174/13816128113199990617. PMID: 24001297.
34. Karim MR, Haque MJ, Akhter S, Ahmed HU. Facebook addiction and its related factors among medical students; a cross- sectional study in Bangladesh. PLOS Glob Public Health. 2023 Feb 22;3(2):e0001597. doi: 10.1371/journal.pgph.0001597. PMID: 36963018; PMCID: PMC10021403.
35. Biggs A, Brough P, Drummond S. Lazarus and Folkman’s psychological stress and coping theory. In: Cooper CL, Campbell Quick J, editors. The handbook of stress and health: a guide to research and practice. John Wiley & Sons; 2017 February. p. 351-64. doi:10.1002/9781118993811.ch21.
36. Compas BE, Jaser SS, Bettis AH, Watson KH, Gruhn MA, Dunbar JP, Williams E, Thigpen JC. Coping, emotion regulation, and psychopathology in childhood and adolescence: A meta-analysis and narrative review. Psychol Bull. 2017 Sep;143(9):939-991. doi: 10.1037/bul0000110. Epub 2017 Jun 15. PMID: 28616996; PMCID: PMC7310319.
37. Carver CS, Connor-Smith J. Personality and coping. Annu Rev Psychol. 2010;61:679-704. doi: 10.1146/annurev.psych.093008.100352. PMID: 19572784.
38. Gross JJ. Emotion regulation: current status and future prospects. Psychol Inq. 2015;26(1):1-26. doi:10.1080/1047840X.2014.940781. Available from: https://www.tandfonline.com/hpli.
39. Skinner EA, Zimmer-Gembeck MJ. The development of coping. Annu Rev Psychol. 2007;58:119-44. doi: 10.1146/annurev.psych.58.110405.085705. PMID: 16903804.
40. Sohail N. Stress and academic performance among medical students. J Coll Physicians Surg Pak. 2013 Jan;23(1):67-71. PMID: 23286627.
