# Supplementary material for: Academic stress and its psychosocial and behavioral determinants in medical students: Findings from a cross-sectional study
Source: PLoS One. 2026 Apr 16;21(4):e0347306. doi: 10.1371/journal.pone.0347306 (PMC13086342; doi:10.1371/journal.pone.0347306)
Supplement: Attachment — Submitted filename: Response to reviewers.docx [file pone.0347306.s007.docx]

**Response to Reviewers**

**Manuscript ID: PONE-D-25-26733**

**Title: Academic Stress and Its Psychosocial and Behavioral Determinants in Medical Students: Findings from a Cross-Sectional Study**

**Dear Editor and Reviewers,**

We sincerely thank the Editor and Reviewers for their careful evaluation and constructive feedback, which have been invaluable in strengthening our manuscript. Their insightful comments guided a comprehensive and systematic revision of the work. In response, we have submitted both a tracked-changes version and a clean version of the manuscript, and have implemented substantial revisions across all sections, from the Introduction through to the Conclusion.

The Introduction has been refined to incorporate relevant evidence from Bangladesh on academic stress, thereby more clearly identifying the existing research gap. It now more effectively highlights the societal relevance and methodological contributions of the study. The Conclusion has also been revised to emphasize the novelty of our findings, their broader implications, and directions for future research. To strengthen the conceptual underpinning, we have integrated Lazarus and Folkman’s stress and coping model into the manuscript. In addition, we have provided a detailed account of the sample size determination process and improved the presentation of results. Specifically, the graphical displays have been revised by replacing violin plots and line charts with median and interquartile range–based summaries, ensuring appropriate representation of the non-normal distribution of the data.

Furthermore, in line with the reviewers’ recommendations, we have adopted more advanced analytical approaches, including Structural Equation Modeling (SEM) and network analysis, and have introduced corresponding network and centrality figures. The reference list has been carefully updated and expanded to reflect the most relevant and recent literature. Additional supplementary materials have been included to enhance methodological transparency, including the rationale for employing a parsimonious latent-variable SEM model, justification for excluding extended sociodemographic covariates, and detailed reporting of model fit indices. A summary table has also been added to clearly demonstrate the triangulation of findings across the different analytical approaches.

Collectively, these revisions have resulted in a more coherent, methodologically rigorous, and substantially strengthened manuscript. We are deeply grateful for the reviewers’ constructive suggestions, which have significantly improved the clarity, rigor, and overall quality of our work. Below, we provide a detailed, point-by-point response to all comments

**Journal Requirement 1**

**Comment:** Ensure manuscript meets PLOS ONE formatting and style requirements.

**Response:** We have revised the manuscript to fully comply with PLOS ONE formatting guidelines, including file naming, structure, and style templates.

**Journal Requirement 2**

**Comment:** Mismatch between Funding Information and Financial Disclosure.

**Response:** We have carefully reviewed and corrected inconsistencies between the Funding Information and Financial Disclosure sections.

“**Financial Disclosure:** This study was conducted as part of the academic curriculum for 4th-phase medical students during the Residential Field Site Training (RFST) program. The study was carried out independently and did not receive any specific grant from funding agencies in the public, commercial, or not-for-profit sectors. No additional external funding was received for this study. “[Line 696-700, p 34].

**Journal Requirement 3**

**Comment:** Amend the funding statement and include the required sentence “Please also include the statement, 'There was no additional external funding received for this study.’ in your updated Funding Statement.

**Response:** The funding statement has been revised to clearly declare all sources of support. We have also included the required sentence: “No additional external funding was received for this study.” [Line 700, p 34].

**Journal Requirement 4**

**Comment:** Clarify the role of funders.

**Response:** We confirm that no funders were involved. The statement has been added:

**“Role of Funders:** The funders had no role in study design, data collection and analysis, decision to publish, or preparation of the manuscript.” [Line 701-3, p 34].

**Journal Requirement 5**

**Comment:** Ethics statement placement.

**Response:** The ethics statement has been moved exclusively to the Methods section and removed from all other sections [Line 211-19, p 10].

**Journal Requirement 6**

**Comment:** Include captions for Supporting Information**.**

**Response:** Captions for all supporting information files have been added and cross-referenced appropriately in the manuscript.

**Editor Comment**

**Comment:** Line 126: Remove link and use proper citation.

**Response:** The hyperlink has been removed and substituted with the standard formula for determining sample size in cross-sectional studies, along with calculations [Line 189-201, p 9].

**Editor Comment**

**Comment:** Close results findings with brackets.

**Response:** All results sections have been revised to ensure proper formatting and closure of statistical reporting.

**Reviewer 1**

**General remark:** “The manuscript addresses a relevant topic and presents two major strengths: an adequate sample size and an interesting set of variables. Considering these aspects, I outline below the main suggestions aimed at achieving a version suitable for publication*.*”

**General response:** Thank you very much for recognizing the relevance of our topic and highlighting the strengths of the manuscript, particularly the adequate sample size and the diverse set of variables. We sincerely appreciate your constructive feedback and detailed suggestions, which have guided us in refining the analytical framework, strengthening methodological rigor, and enhancing the clarity of our revision. Your comments have been invaluable in improving the overall quality and publication readiness of the manuscript.

**Comment 1.1 — Introduction: Include data on Bangladesh about the variables investigated in the study.**

**Response:** We have expanded the Introduction to include additional, Bangladesh‑specific evidence on prevalence and correlates of academic stress, depression, anxiety, sleep disturbance, and internet use among tertiary and medical students. We cite national studies and briefly summarize their findings to situate our work in the local context.

**Revision made:** **Introduction** — added one paragraph summarizing prior Bangladeshi studies and their findings (new text added to the end of the original Introduction). These paragraphs explicitly reference the multicenter and single‑site studies cited in the original manuscript and clarify how our study extends that evidence. [line 100-109, p 5]

**Comment 1.2 — Introduction/Discussion: Data collection coincided with the COVID‑19 pandemic; this needs to be considered.**

**Response:** We acknowledge the timing of data collection and its potential influence on psychological and behavioral measures. We added a dedicated paragraph in the Methods describing the data collection period (October–December 2022). In the Discussion, we explicitly consider how pandemic-related disruptions and residual effects (e.g., altered learning formats, social restrictions) may have influenced prevalence estimates and behavioral patterns.

We also discuss the direction and magnitude of potential bias and note that the multicenter sampling and validated instruments mitigate, but do not eliminate, this concern.

**Revision made:** **Introduction-** Covid-19 effect on academic stress [Line 110-12, p 5]; **Methods (Data collection subsection)** — clarified dates and contextualized them with respect to the pandemic [Line 234-39, p 11]; **Discussion** — added explanation discussing possible pandemic effects and how they were considered in interpretation [Line 647-55, p 31-32].

**Comment 1.3 — Clarify Stress Coping Style Inventory subscales and whether strategies are dysfunctional.**

**Response:** We added a concise description of each SCSI subscale (Active Problem Coping, Active Emotion Coping, Passive Problem Coping, Passive Emotion Coping), clarifying which strategies are generally considered adaptive versus maladaptive in the literature and how we interpret higher scores on each subscale in relation to academic stress. This clarification prevents misinterpretation of the coping results in the Discussion.

**Revision made:** **Methods (Measures subsection: Stress Coping Style Inventory)** — added description defining each subscale and indicating typical adaptive/maladaptive classification [line 248-56, p 11-12]; **Discussion** — referenced these definitions when interpreting coping results [line 616-21, p 30].

**Comment 2.1 — Methods: See suggestions 5.4.1, 5.4.2, and 5.5 (analytical precision).**

**Response:** We implemented the recommended advanced analyses to address the complexity of relationships among variables: (a) Structural Equation Modeling (SEM) to test the hypothesized mediated pathways with psychological distress as a latent construct and behavioral variables as mediators; and (b) Network Analysis to visualize and quantify interdependencies and centrality of variables. Both analyses were added to the manuscript, and their results were integrated with the logistic regression findings. These additions directly address the reviewer’s concern about overly simplistic analysis.

Multiple SEM models were evaluated to determine the best fit based on global fit measures and indices. This included comparisons between the latent-only model and an extended model that incorporated sociodemographic covariates, as well as a parsimonious stress-coping model versus a segregated stress-coping sub-scales integrated model. To maintain rigorous statistical reporting standards, we focused on presenting the latent-only model and the stress-coping scale inventory (SCSI) parsimonious model; however, the extended models are included in the supplementary files.

**Revision made:** **Methods (Statistical analysis subsection)** — expanded to describe SEM specification (latent PSY indicated by PHQ‑9 and GAD‑7; mediators: ISI (sleep quality, SQ); IAT (internet addiction); RSES (self-esteem); SCSI (stress coping style inventory) subscales; sociodemographic covariates included as exogenous controls) and network analysis procedures (estimation method, centrality metrics) [Line 307-27, p 14-15] **Supplementary Materials (S1, S2, S3, S4)**.

**Results** — added SEM fit indices and path coefficients; added network graph and centrality table (new Figure and Table). **Supplementary Materials (S1, S2, S3, S4)** — provided full SEM diagrams and network plots [Line 406-539, p 20-26].

**Comment 3.1 and 3.2 — Results: In logistic regression, were all variables entered simultaneously? Clarify control variables.**

**Response:** Yes. We clarified that the binary logistic regression used the Enter method, with all candidate predictors entered simultaneously to estimate adjusted effects. We explicitly list the control variables (sex, age, residence, family type, parental education, monthly expenditure, and academic year) and indicate how categorical variables were dummy coded. We also report multicollinearity diagnostics (VIF) and model fit statistics.

**Revision made:** **Methods (Statistical analysis subsection)** — added explicit statement that the Enter method was used and listed control variables and coding; [Line 301-3, p 14].

**Results (Regression subsection)** — added VIF results and model fit information; **Table** — updated to show adjusted odds ratios (AOR), 95% CIs, p‑values, and footnotes explaining the model fit [Line 420-24, p 20-21; Table 3, p 23].

**Comment 4.5 — Use theory and prior evidence to justify SEM and moderator/mediator roles.**

**Response:** We expanded the Theoretical Framework section to more explicitly link the Transactional Model of Stress and Coping to our SEM specification. We justify the selection of mediators (sleep disturbance, internet addiction, self‑esteem, coping) and the inclusion of sociodemographic variables as exogenous controls and potential moderators, citing prior empirical studies that support these roles. Moderator tests were performed where theoretically justified (e.g., testing whether residence or academic year moderated the PSY → academic stress path). Results of the moderation tests are reported.

**Revision made:** **Theoretical Framework** — added explicit rationale and citations for mediator/moderator assignments [142-69, p 7-8]; **Methods (SEM subsection)** — described moderator tests [Line 307-9, p 14]; **Results** — reported moderation test outcomes [Line 478-89, p 24; Line 516-23, p 25, Figure 1, Figure 4, Figure 5, Table 4, p-26].

**Comment 5.1 — Language and style: Orthographic and technical revision of the manuscript: apply more technical and objective language.**

**Response:** We performed a comprehensive language edit to improve technical tone and clarity. Examples provided by the reviewer were rephrased using more objective scientific language throughout the manuscript.

**5.1.1**- Forest plot removed to avoid duplicating the logistic regression findings. Other important figures, such as Figure 4, Figure 5, and supplementary materials (S1, S2, S3, S4), illustrate the findings with clarity and precision.

**5.1.2**- revised as recommended [Line 556-63, p 27]

**5.1.3**- revised a recommended [Line 588-96, p 29]

**Revision made:** Full manuscript revised for language and style; specific sentences reworded as suggested (see tracked changes in the submitted revision).

**Comment 5.2 and 5.3 — Figures and tables quality/formatting.**

**Response:** We improved figure resolution and formatting, standardized fonts and labels, and corrected table formatting issues in the lower sections. All figures were regenerated for clarity; tables were reformatted to ensure consistent alignment and legibility.

**Revision made:** **Figures 1–5 and Tables 1–4** — replaced with higher‑resolution versions and corrected formatting; figure captions expanded for clarity.

**Comment 5.4 [5.5.1, 5.4.2] & 5.5 —** We appreciate the reviewer’s constructive observation regarding the analytical precision of our study. In line with the suggestions, we have substantially strengthened the analytical framework:

**Response: Structural Equation Modeling (SEM):** We implemented SEM to test an explanatory model guided by the Transactional Model of Stress and Coping. Psychological distress (latent construct indicated by depression and anxiety) was modeled as the independent variable, academic stress as the dependent variable, and behavioral factors (sleep disturbance, internet addiction, self‑esteem, coping styles) as mediators. Sociodemographic variables were incorporated as exogenous controls and, where theoretically justified, as moderators. Fit indices (CFI, RMSEA, SRMR) and indirect effects (bootstrap estimates) are reported, allowing us to rigorously assess both direct and indirect pathways.

**Two Lavaan syntaxes were used**

**Latent-only SEM model in lavaan model <- '**

\# 1. Measurement Model

PSY =~ GAD_total + PHQ_total

\# 2. Structural Model

\# Direct effect of PSY on academic stress

AS_total ~ c*PSY

\# Mediator regressions

SQ_total ~ a1*PSY

SE_total ~ a2*PSY

IAT_total ~ a3*PSY

SC_total ~ a4*PSY

\# Academic stress predicted by mediators

AS_total ~ b1*SQ_total + b2*SE_total + b3*IAT_total + b4*SC_total

\# 3. Indirect Effects

ind_SQ := a1*b1

ind_SE := a2*b2

ind_IAT := a3*b3

ind_SC := a4*b4

total_ind := ind_SQ + ind_SE + ind_IAT + ind_SC

\# 4. Measuring covariances

GAD_total ~~ PHQ_total

\# 5. Total Effect

total := c + total_ind

**Extended Measurement Model including sociodemographic controls**

\# Latent Psychological Distress

PSY =~ GAD_total + PHQ_total

\# 2. Structural Model

\# Direct effect

AS_total ~ c*PSY

\# Mediator regressions

SQ_total ~ a1*PSY

SE_total ~ a2*PSY

IAT_total ~ a3*PSY

SC_total ~ a4*PSY

\# Academic stress predicted by mediators

AS_total ~ b1*SQ_total + b2*SE_total +

b3*IAT_total + b4*SC_total

\# 3. Sociodemographic Controls

AS_total ~ Live_area_2cat + Mother_edu_2 + Famtype_2cat +

Expen_cat + Sex

SQ_total ~ Ad_yr + Sex

SE_total ~ Ad_yr + Sex

IAT_total ~ Ad_yr + Sex

SC_total ~ Ad_yr + Sex

\# 4. Indirect Effects

ind_SQ := a1*b1

ind_SE := a2*b2

ind_IAT := a3*b3

ind_SC := a4*b4

total_ind := ind_SQ + ind_SE + ind_IAT + ind_SC

GAD_total ~~ PHQ_total

\# 5. Total Effect

total := c + total_ind

[Line 407-19, p 20; Line 476-89, p 24]; Figure 4; S1 & S2.

**5.4.2- Network Analysis:** We conducted network analysis to visualize and quantify the multivariate interdependencies among all observed variables. Centrality metrics (Strength, Closeness, Betweenness, Expected Influence) were estimated, enabling identification of the most influential variables within the system. This analysis provided complementary insights to SEM, highlighting bridging roles (e.g., sleep disturbance) and the strong centrality of depression and internet addiction. [Line 501-6, p 25]; Figure 5, Table 4, S3 & S4.

**5.5- Complex Interplay Claim:** With the addition of SEM and network analysis, our statement that “this study highlights the complex interplay of factors” is now empirically substantiated. The integrated findings demonstrate that psychological distress exerts both direct and mediated effects on academic stress, while behavioral and sociodemographic variables shape these pathways in nuanced ways. The network analysis further confirms the interconnectedness of variables, offering a richer understanding than regression alone. [Line 516-23, p 25], Table 4.

**Revisions made: Methods (Statistical Analysis section):** Expanded to describe SEM specification and network analysis procedures [Line 307-27, p 14-15].

**Results:** Added SEM fit indices, path coefficients, indirect effects, and network centrality metrics [Line 406-539, p 20-26].

**Figures/Tables:** Included SEM diagram [Fig 4] and network graph [Fig 5]; added synthesis table [Table 4], S1, S2, S3, S4.

**Discussion:** Revised to interpret SEM and network findings, linking them to theory and prior evidence [Line 626-46, p 30-31].

We thank the reviewer for these insightful recommendations, which have significantly enhanced the technical rigor and validity of our conclusions

**Comment 5.6 — Practical implications section requested.**

**Response:** We revised the conclusions and recommendations section, adding a paragraph on practical implications, followed by specific recommendations. This section outlines concrete institutional and curricular interventions (mental health screening and referral pathways, sleep hygiene programs, digital‑use education, coping‑skills workshops), prioritized according to the strength of associations observed (e.g., targeting depression/anxiety and problematic internet use). Each recommendation is linked to the relevant empirical finding.

**Revision made:** **Conclusion and recommendations (Practical Implications)** — new content with prioritized, actionable recommendations. [Line 674-90, p 33].

**Reviewer 2**

**General remark:** *Commendation and suggestions to strengthen societal and methodological contributions, emphasize sample strengths, and refine the conclusion and recommendations.*

**Comment 2.1 —** “While the gap in the literature is clearly articulated, I encourage you to expand the justification for the study by more explicitly highlighting its social and methodological contributions. Beyond addressing an academic gap, what are the broader societal implications of your findings? How might they inform institutional policies, mental health interventions, or educational reforms? Additionally, clarifying the methodological contributions of the study (such as the integration of multiple predictors within a single analytical framework) would further reinforce the originality and added value of the research. Strengthening these aspects would enhance the overall rationale and impact of the manuscript**.”**

**Response:** We strengthened the Introduction, method, result, and Discussion to more explicitly articulate the broader societal implications (impact on future healthcare workforce, institutional policy relevance) and the methodological contributions (large, geographically representative sample; integration of multiple predictors using SEM and network analysis). We added a short paragraph in the Introduction that frames how findings can inform policy and institutional practice.

**Revision made:** **Introduction** — added paragraph on societal and policy relevance [Line 117-31, p 6]; **Methods (Statistical analysis)** – expanded and explained in method section [Line 307-27.p14-15]; **Results** – Thoroughly revised in accordance with the gaps, objectives, and theory [Line 406-523, p 20-25], Table 4, S1, S2, S3, S4; **Conclusion & recommendations** — expanded to highlight methodological contributions and how they improve inference and intervention prioritization [Line 667-92, p 32-33].

**Comment 2.2 — “**The study benefits from a large and well-distributed sample, which significantly strengthens the robustness of the analyses and the generalizability of the findings. This is an important methodological strength and should be clearly emphasized as such in the manuscript.”

**Response:** We added a clear statement in Methods and the Discussion emphasizing the multicenter, stratified random sampling across eight divisional medical colleges and how this enhances representativeness and generalizability to public medical students in Bangladesh. We also discuss limits to generalizability (e.g., private colleges not sampled).

**Revision made:** **Methods (Sampling subsection)** — clarified sampling rationale and representativeness [Line 182-210, p 8-9]; **Discussion (Strengths and Limitations)** — emphasized generalizability and boundaries. [Line 657-60, p 32].

**“Results:** The results are presented in a clear, coherent, and methodologically appropriate manner. No additional comments or revisions are necessary.”

**Response:** acknowledged with thanks.

**“Discussion:** No additional comments. I consider this section to be very well written, coherent, and appropriately supported by the literature.”

**Response:** acknowledged with thanks.

**Comment 2.3 —** “Conclusion and Recommendations: I recommend strengthening this section by emphasizing the broader scientific, educational, and social significance of the study. Rather than reiterating previously presented results at the outset (which makes the section somewhat repetitive) it would be more impactful to focus on the implications of the findings for medical education, mental health policy, and institutional practices. If the authors consider it essential to retain a concise summary of the main findings, I suggest relocating this synthesis to the end of the Discussion section, where it would serve as a natural transition into the conclusion. This adjustment would improve the manuscript’s structure, reduce redundancy, and enhance the overall coherence and flow of the paper. It would be valuable to include explicit recommendations for future research based on the limitations identified in the present study. Doing so would not only strengthen the scientific contribution of the manuscript but also provide meaningful guidance for future researchers in this field. Clearly outlining potential directions (such as longitudinal designs, inclusion of additional psychosocial variables, or cross-cultural comparisons) would enhance the article’s impact and relevance for advancing the literature on academic stress.”**:**

**Response:** We thank the reviewer for this insightful recommendation. In line with the suggestion, we have revised the Conclusion to emphasize the broader scientific, educational, and social significance of the study, focusing on implications for medical education, institutional practices, and mental health policy rather than repeating results.

To improve coherence, the concise summary of findings has been relocated to the end of the Discussion, serving as a natural transition into the Conclusion.

Additionally, we have included a dedicated Practical Implications statement under the Conclusion, highlighting concrete pathways for intervention. Specifically, we recommend:

- Integrating routine mental health screening and counseling services into medical colleges.
- Promoting sleep hygiene and responsible internet use through structured awareness programs.
- Embedding coping‑skills training into the curriculum to strengthen resilience.
- Developing institutional policies that prioritize student well‑being alongside academic performance.

We have also added explicit recommendations for future research, including longitudinal designs, incorporation of additional psychosocial variables, and cross‑cultural comparisons, to advance the literature on academic stress.

**Revisions made:** **Discussion:** Summary of findings moved to the end; expanded with future research directions. [Line 626-46, p 30-31].

**Conclusion and recommendations:** Rewritten to emphasize broader implications and now includes a clear Practical Implications subsection with prioritized recommendations. [Line 674-84], p 33; Line 685-92, p 33].

We appreciate the reviewer’s guidance, which has significantly improved the structure, clarity, and impact of the manuscript

**Comment 2.4 — Proofreading and adherence to journal guidelines.**

**Response:** The manuscript underwent careful proofreading for spelling, grammar, and formatting. We also verified compliance with the journal’s submission guidelines and updated the manuscript file and supplementary materials accordingly.

**Revision made:** Full manuscript proofread and formatted to journal specifications; updated cover letter and checklist included with resubmission.

**Key methodological clarifications requested by reviewers (consolidated)**

**A. SEM and Network Analysis implementation details** **Response:** We provide the following concise technical details in the revised Methods and Results:

**SEM:** Psychological distress (PSY) specified as a latent variable indicated by PHQ‑9 and GAD‑7; mediators: ISI (sleep disturbance), IAT (internet addiction), RSES (self‑esteem), and SCSI subscales (coping). Sociodemographic variables were entered as observed exogenous covariates. Model fit indices reported: CFI, RMSEA, SRMR, and χ²/df. Indirect effects estimated using bias‑corrected bootstrap (5,000 samples).

**Network analysis:** Estimated using Gaussian graphical models with regularization (EBICglasso). Centrality metrics reported: Strength, Closeness, Betweenness, and Expected Influence. Stability and accuracy assessed via bootstrapping.

**Revision made:** **Methods (Statistical analysis subsection)** — added these technical details; **Results** — added SEM with standardized coefficient, fit indices, and network centrality; **Supplementary Materials** — full SEM diagram and network plots with bootstrap results.

**B. Regression modeling details and multicollinearity checks** **Response:** We clarified that the Enter method was used for logistic regression, listed all covariates, and reported VIFs (all < 2.5), indicating acceptable multicollinearity. Model calibration (Hosmer‑Lemeshow) and discrimination (AUC) statistics are reported.

**Revision made:** **Results (Regression subsection)** — All variables significant in bivariate analysis with crude ORs were simultaneously entered into the multivariable logistic regression model, and the table now presents AORs with 95% CIs along with model diagnostics (VIFs, Hosmer–Lemeshow p‑value, and AUC) clearly explained in the footnotes.

**Minor and editorial changes**

- Corrected figure and table formatting issues and improved figure resolution.
- Reworded sentences flagged by Reviewer 1 to adopt more technical phrasing.
- Added a short Limitations paragraph addressing cross‑sectional design, self‑report measures, and pandemic timing.
- Added a Practical Implications subsection with prioritized, actionable recommendations.
- Updated references and ensured all psychometric instruments cited with appropriate validation studies for Bengali adaptations.

**Summary of major additions (for quick reference)**

- **SEM and Network Analysis** were added and integrated with logistic regression results (Methods; Results; Supplementary Materials).
- **Expanded Introduction** with Bangladesh‑specific evidence and clearer statement of societal/methodological contributions.
- **Pandemic timing** is explicitly acknowledged and discussed.
- **Coping subscales** defined and interpreted.
- **Practical Implications** subsection added with prioritized interventions.
- **Language and formatting** improvements; figures and tables reformatted

Dr Md. Rizwanul Karim
